# Social working memory in adolescence

**DOI:** 10.1093/chidev/aacaf039

**Published:** 2026-01-29

**Authors:** Karina Grunewald, Jack L Andrews, Susanne Schweizer

**Affiliations:** School of Psychology, University of New South Wales, Sydney, Australia; Department of Experimental Psychology, University of Oxford, Oxford, United Kingdom; School of Psychology, University of New South Wales, Sydney, Australia

**Keywords:** adolescence, working memory, social network

## Abstract

Little is known about how social network associations are tracked in cognition during adolescence, when social networks change in size and complexity. In 2023, 123 ethnically diverse adolescents and emerging adults (13–24 years; 52.9% female; 68.3% White; 18.7% Asian) completed a task measuring working memory (WM) differences for social and nonsocial network information. Additionally, this sample was combined with an existing sample recruited in the same year (*N* = 241, 18–65 years; 59.3% female; 64.7% White; 16.6% Asian) to investigate age-related differences in social and nonsocial WM performance. A WM advantage for social over nonsocial networks was observed across adolescence, emerging adulthood, and adulthood, especially for self-relevant social information (*R*^2^ = 0.01–0.02). Age was also positively associated with WM performance. Findings provide insights into how individuals learn about social relationships in adolescence and emerging adulthood, the successful formation of which has lasting impacts on wellbeing.

Adolescence (10–24 years; Sawyer et al., 2018) is marked by wide-ranging social changes, including more time spent with peers and greater susceptibility to peer influence (Guyer et al., 2016; Knoll et al., 2015; Nelson et al., 2016). Adolescents must learn to navigate their expanding and increasingly complex social environments. To do so they rely on a number of socio-cognitive abilities (Andrews et al., 2021), including social working memory—the ability to retain and manipulate limited amounts of social information in mind (Meyer et al., 2012). Adults show a clear working memory advantage for social (e.g., human social networks) over nonsocial (e.g., transport networks) network information (Andrews et al., 2024). However, little is known about when in development this advantage emerges, with working memory capacity still developing in adolescence (Tervo-Clemmens et al., 2023). The current study therefore aimed to investigate whether adolescents also show a social working memory advantage.

Developmentally, adolescence is a period of life marked by continued changes in social cognition, characterized by improved processing of faces compared to childhood (Cheng et al., 2024), and preferential processing of social information (Andrews et al., 2019, 2021; though see: De Lillo et al., 2021). This heightened social sensitivity may potentiate the working memory advantage for social over nonsocial information observed in adults (Andrews et al., 2024). In addition to developmental differences in the sensitivity to social information, individuals with high levels of depressive symptoms also demonstrate heightened sensitivity to social evaluation (Gao et al., 2017). This sensitivity is expressed in a hypervigilance to social evaluation, which is proposed to lead to prioritized processing of social, especially negative social information (Allen & Badcock, 2003). Depression symptoms then may interact with development to significantly impact adolescents’ ability to learn about and successfully navigate social environments.

To investigate age- and depression-related differences in social working memory, the current study administered the network memory task (NMT; Andrews et al., 2024) to 123 adolescents. The NMT assesses participants’ working memory for nonsocial (i.e., flightpaths between airports) and social (i.e., friendships) network ties. In the task, half the social networks include the “self” and the other half include only “others”. Self-relevance has been shown to influence memory in adolescence (Dégeilh et al., 2015; Sweatman et al., 2022) and neuroimaging evidence shows differential processing of self- and other-related information in adolescence (e.g., van Buuren et al., 2020). However, whether a self-relevance effect can be observed for working memory in adolescence remains untested.

In adults, working memory in general, and social information in particular, has been shown to vary across valence (Schweizer et al., 2019). Psychologically healthy individuals are more likely to remember positive self-relevant information (Leshikar et al., 2015), whereas individuals who experience symptoms of depression are more likely to remember negative self-relevant information (Bone et al., 2021). However, the self-reference effect has been predominantly investigated in relation to episodic memory. Interestingly, in a previous study using the NMT to investigate social working memory, an observed self-reference effect varied across valence, with a positivity bias in working memory for social ties from networks including the self but a negativity bias in working memory for networks including only others (Andrews et al., 2024). The same self-referent positivity bias, however, may not be observed in adolescence, when sensitivity to social exclusion is heightened (Andrews et al., 2022). In fact, working memory may be better for negative compared to positive self-relevant social information in adolescents, as evidence from the episodic memory literature shows that while the memory positivity bias remains stable across adolescence for self- and other-relevant information, the negativity bias decreases selectively for self-relevant information (Moses-Payne et al., 2022). Using the NMT in an adolescent sample allowed us to test the effect of valence on adolescent social working memory. Specifically, the current study examined the following preregistered hypotheses:

The recall of social compared to nonsocial network associations would be faster and more accurate (H1a), especially for networks including the self (H1b); and these effects (H1a and H1b) would be stronger for working memory of negative compared to positive network ties (H1c). Social sensitivity was predicted to be positively associated with faster and more accurate working memory for social compared to nonsocial information (H2a), especially for networks including the self (H2b) and for information about negative social ties (H2c). We also predicted that these effects (H1 and H2) would be more pronounced in adolescents compared to adults (H3). To test this third hypothesis, we combined data from adolescents (13–24 years) with a second dataset including adolescents and adults (18–65 years) from a previous study using the same task (Andrews et al., 2024). Finally, the social working memory advantage (H1a) was hypothesized to be associated with better social functioning in adolescents (i.e., increased network size, quality, and satisfaction; H4).

## Method

This study was approved by the University of New South Wales Human Research Executive Committee (HC Number 220233) and was preregistered prior to participant recruitment (https://osf.io/brnxc).

### Participants

As per the preregistration, participants were recruited between January and June 2023, until 128 unique participants completed the study faithfully (passed at least 3 out of 5 attention check items included throughout the experiment). A sample size of 116 was calculated to have a power of 0.95 to detect an interaction effect of age (predicted to be small to be conservative, *f* = 0.12) with sociality (social-self, social-other, nonsocial), valence (negative, positive), and depressive symptoms (which showed a small-moderate *f* = 0.17 interaction effect; https://osf.io/4fcxn). The combined effect size ((0.17 * 0.12)/2) *f* = 0.145 was entered into the power calculation with a significance threshold of *α* = .05. To account for attrition with online experiments, we aimed to increase the sample size by 10%. Participants were recruited through advertising on the MQ (charity) participate platform, social media, and Prolific. Where possible, advertisements were targeted at individuals residing in Australia or the United Kingdom.

To participate in the present study, participants had to be 13–24 years of age, fluent in English, as the NMT relies on adequate reading ability, and have no history of head injury, neurological disorder, or diagnosed learning disability. For further details on participant inclusions, exclusions, and sample characteristics, see supplementary materials. The final sample comprised 123 participants (60 adolescents; 63 emerging adults; Table S1A). While adolescence is recognized as the period between 10 and 24 years of age (Sawyer et al., 2018), here we further differentiated our sample between adolescents (13–17 years) and emerging adults (18–24 years). Emerging adults here refers to the period of life when adolescents begin the transition to adulthood (Arnett, 2000, 2007). To test hypothesis 3, this sample was combined with 241 participants (aged 18–65 years) from a previous study using the same task (Andrews et al., 2024; Table S1B). Participants aged 18–24 years were added to the current emerging adult sample, and participants aged 25–65 years made up the adult sample. This resulted in 60 adolescents, 111 emerging adults, and 193 adults included in the analyses for hypothesis 3.

Participants who completed the study faithfully were reimbursed the standard departmental rate of AUD20 or £10 for their participation in the study through a Prezzee voucher or Prolific compensation.

### Procedure

After providing informed consent, or assent and parental consent for participants under 18 years, participants completed a set of questionnaires, followed by the NMT. Altogether, the study took less than 1 hr to complete. All study components were completed online on the Gorilla experimental platform.

#### Questionnaires

Participants completed standard demographics questions, including age, gender, and ethnicity.

##### Depressive symptoms

To measure symptoms of depression, participants completed the depression subscale of the short Depression Anxiety and Stress Scale (DASS-21; Lovibond & Lovibond, 1995). The scale demonstrated good internal consistency in the current sample (*ω*_T_ = .96).

##### Social sensitivity

Social sensitivity was assessed with the Online and Offline Social Sensitivity Scale (O^2^S^3^; Andrews et al., 2022), which also demonstrated good internal consistency in the current sample (*ω*_T_ = .93).

##### Personal social networks

To assess *social network size*, participants were asked to report the number of friends they interact with regularly offline and online (e.g., “How many friends do you have that you see regularly in person?”). For online networks, participants were also asked to report the number of people they “followed” and that were “following” them on their preferred social media platform. A *cool ratio* (Moss, 2014) was computed as the ratio of the number of people participants follow on social media to the number of people that follow them. *Social network quality* was indexed as the number of friends they would trust to keep a secret and could ask a favor of (e.g., “How many friends do you have you would trust to keep a secret for you?”). Participants entered their responses to each of these questions into text boxes, with no limit on the number of friends they could report for each. To measure *social network satisfaction,* participants rated how happy they were with how often they interacted with their friends online and in-person, on two separate visual analogue scales ranging from 0 (very unhappy) to 100 (very happy).

#### Network Memory Task

**Figure 1:**
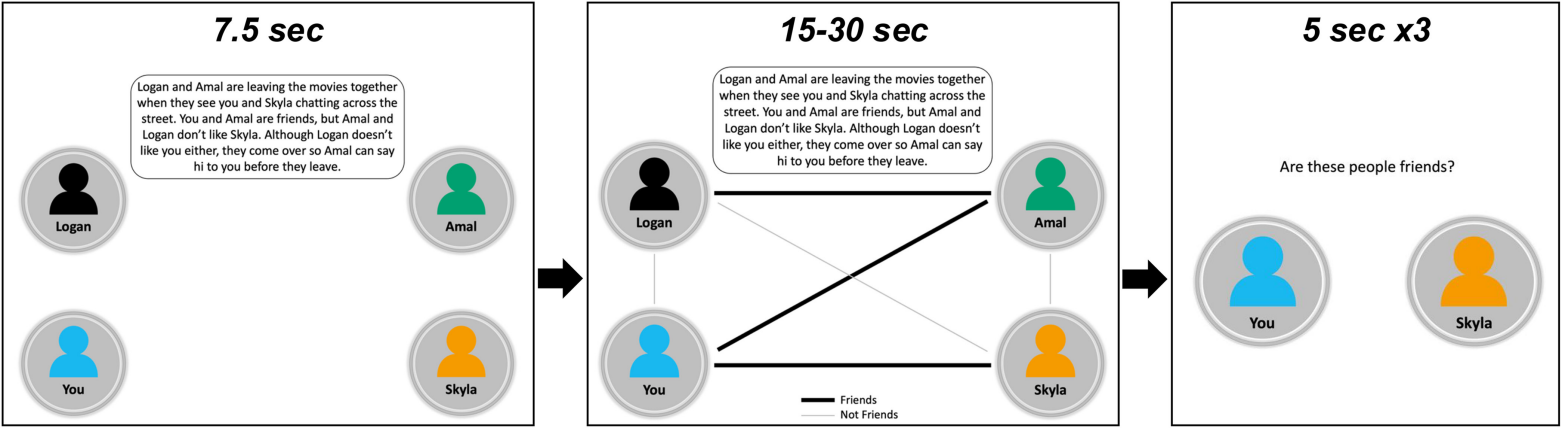
Network memory task (NMT). *Note*. A schematic overview of the NMT (social-self condition). Participants saw a vignette describing the relationships within a network for 7.5 s, followed by a network diagram of these relationships shown for an additional 30 s (with option to proceed after 15 s). Participants then saw three individual connections from the network and indicated whether the people shown were friends (social conditions) or flights shown were running (nonsocial condition). An exploratory sensitivity analysis investigating the effect of reading time (seconds) by condition (nonsocial: *M* = 18.89, *SD* = 2.28; social-other: *M* = 19.02, *SD* = 2.06; social-self: *M* = 18.90, *SD* = 2.25) yielded no significant effect (*F* = 0.30, *df* = 239.27, *p* = .742).

The NMT (Figure 1; Andrews et al., 2024) included three conditions that were presented in counterbalanced order across participants: *social-self* (a social network including the self), *social-other* (a social network including only others), and *nonsocial* (a network of flights between cities). Each condition included 12 networks, with half the relationships in the networks being positive and half negative. Working memory load varied across trials, with half the networks including three items and the other half including four items (individuals or flight connections). For each network, ­participants read a vignette describing the relationships within the network. After 7.5 s, a network diagram illustrating these associations appeared for up to 30 s (self-timed progression 15 s after the diagrams appear). Participants were then asked to classify three connections (i.e., 36 connections per condition) from each network as positive or negative as quickly as possible. Working memory performance was operationalized as reaction time on correct trials and accuracy (total number of correct trials).

### Analyses

All statistical analyses were conducted using R version 4.2.1. All reported statistics were based on linear mixed models including participant ID as a random intercept, unless otherwise specified. As recommended for mixed-effect models, effect sizes were calculated using r squared (Nakagawa & Schielzeth, 2013).

Hypotheses 1a and b were tested with condition entered as a predictor of reaction time (RT) and accuracy. Hypothesis 1c was tested by adding valence (negative vs. positive) as a predictor to the models testing H1a and H1b.

To test hypotheses 2a–c, social sensitivity (indexed as the total score on the O^2^S^3^) was added as a predictor to the models specified for H1a–c. As per the preregistration, all predictions made under H2 for social sensitivity were also tested for depressive symptoms (indexed as the total score on the depression subscale of the DASS-21) in exploratory analyses.

To test hypothesis 3, the present sample was combined with a sample from a previous study (Andrews et al., 2024). The total sample size for the H3 analyses was *N* = 364 (for characteristics of the full sample, see Table S1B). After combining the two samples, participants were divided into three age groups: adolescents (13–17 years, *n* = 60), emerging adults (18–24 years, *n* = 111), and adults (25–65 years, *n* = 193). Age (adolescent vs. emerging adult vs. adult) was then added as a predictor to the models testing hypotheses 1 and 2.

To test hypothesis 4, size (the sum of the total number of friends regularly seen in person and online) quality (the sum of the total number of friends trusted to keep a secret and do a favor) and satisfaction (the sum of satisfaction with how often they see friends in person and online) of participants’ personal social networks were separately added as predictors to the model testing H1a.

### Data and code availability

On publication of the manuscript, de-identified data and analytic code necessary to reproduce the analyses presented in this paper will be publicly accessible at the Open Science Framework (https://osf.io/brnxc).

## Results

### Social working memory advantage

**Figure 2:**
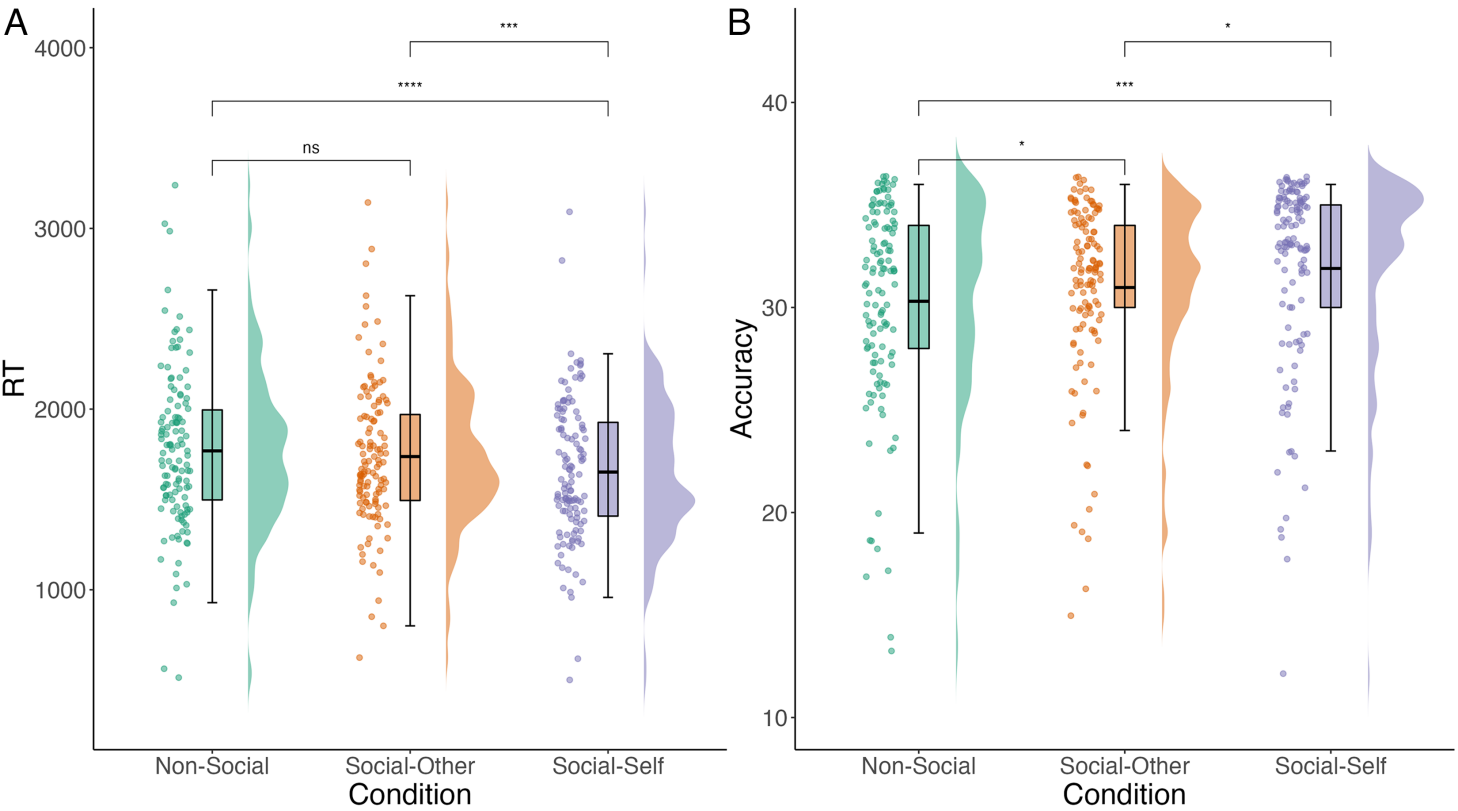
Network task performance depending on condition*. Note*. (A) Adolescent reaction time performance across network task conditions (nonsocial vs. social-other vs. social-self); reaction time is operationalized as average reaction time on correct trials of the network task. (B) Adolescent accuracy across network task conditions (nonsocial vs. social-other vs. social-self); accuracy is operationalized as number of total correct trials out of 36.

As predicted, we observed a self-reference effect in working memory, evidenced by a significant main effect of condition as a three-level factor (social-self, social-other, nonsocial) (RT: *F* = 13.74, *df* = 244.00, *p* < .001; accuracy: *F* = 8.82, *df* = 244.00, *p* < .001; Table S3B). Pairwise comparisons (Table S4) demonstrated that adolescents recalled network information significantly faster (Figure 2A) and more accurately (Figure 2B) for networks that included the self, compared to social networks comprised of others and nonsocial networks. Although we observed an overall working memory advantage for the recall of social network connections (RT: *F* = 13.04, *df* = 245.00, *p* < .001; accuracy: *F* = 11.55, *df* = 245.00, *p* = .001; see Table S3 for full model statistics), no significant difference was found for speed or accuracy of network recall between social networks comprised of others and nonsocial networks (Table S4).

Together, these results indicate, as predicted, that adolescent working memory performance was fastest and most accurate for social compared to nonsocial relational information, especially for social information pertaining to the self.

### Working memory for positive and negative network ties

**Figure 3:**
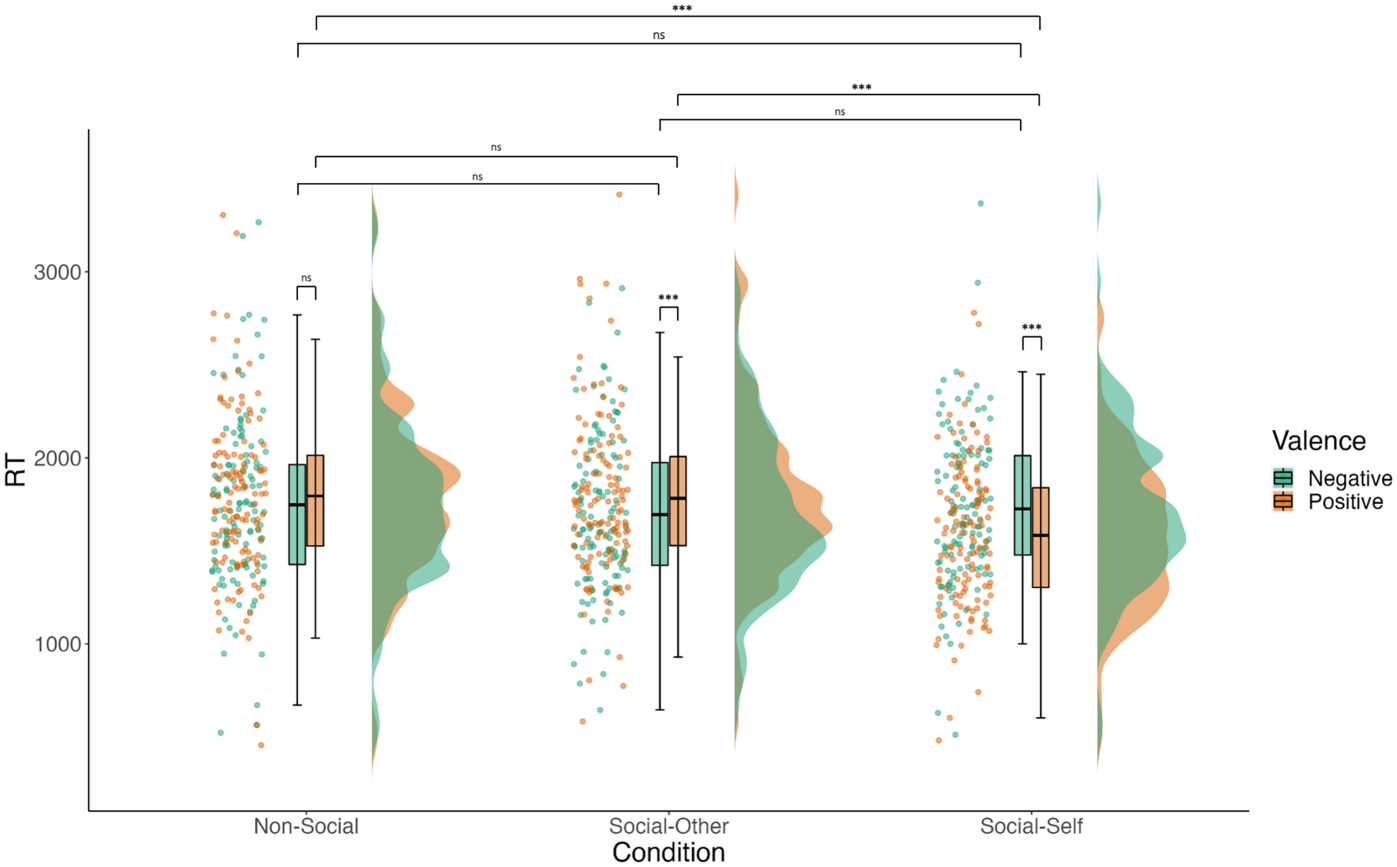
Network task performance depending on valence and condition*. Note*. Interaction effects between valence (negative vs. positive) and condition (nonsocial vs. social-other vs. social-self) on adolescent reaction time in the network task. Reaction time is operationalized as average reaction time on correct trials of the network task. Negative valence refers to trials that tested working memory for associations between individuals who are not friends/flights that are not running between cities, whereas positive valence refers to trials that tested working memory for associations between individuals who are friends/flights that are running between cities.

When examining whether working memory performance for negative compared to positive associations differed across social and nonsocial networks (Table S3C), in partial support of our second hypothesis, valence (negative vs. positive) significantly interacted with condition (social vs. nonsocial) for RT (*F* = 4.66, *df* = 611.05, *p* = .031), but not accuracy (*p* = .431). The same was true for condition when comparing across the nonsocial, social-other, and social-self conditions: (RT: *F* = 19.74, *df* = 609.02, *p* < .001; Accuracy: *p* = .724; Table S3D). Pairwise comparisons showed no significant effects of valence for working memory in the nonsocial and social conditions (Table S5A). This replicated previous findings showing that this was due to opposing effects in the social-self versus social-other conditions (Table S5B; Andrews et al., 2024). Specifically, adolescent working memory performance was faster for negative compared to positive associations in social networks including only others, while positive ties were remembered faster in social networks including the self (Figure 3).

### Social working memory advantage across low and high cognitive load

In response to reviewer comments, we ran exploratory analyses investigating differences in the social working memory advantage across low and high cognitive load. We observed a general effect of load on network task performance (Table S12), with participants recalling network information faster (*F* = 306.73, *df* = 614.00, *p* < .001) and more accurately (*F* = 77.21, *df* = 614.00, *p* < .001) in low (*M* = 16.15, *SD* = 2.47) compared to high (*M* = 14.91, *SD* = 2.84) load trials (Figure S4). We also observed significant interactions between load and condition on task performance, with load showing the greatest effect in the social-self and nonsocial conditions (Table S13; Figure S5). In low load trials, participants accurately recalled on average approximately 16 nonsocial, 16 social-other, and 17 social-self ties (out of 18 total ties in each condition). In high load trials, participants accurately recalled on average 14 nonsocial, 15 social-other, and 15 social-self ties (out of 18 total ties in each condition).

### Social sensitivity and social working memory advantage

Neither the social working memory advantage (Table S6A) nor the social self-relevance effect (Table S6B) increased as a function of social sensitivity. Moreover, social sensitivity did not interact with valence across the social and nonsocial conditions to predict adolescent working memory performance (Table S6C, D).

Excessive social sensitivity is a risk factor for depressive symptoms and has been proposed to account for the heightened vulnerability for the onset of depressive symptoms during adolescence. We therefore preregistered exploratory analyses to investigate the impact of depressive symptoms across social and nonsocial adolescent working memory (Table S7). Results showed that when controlling for valence, reaction time in the social versus nonsocial condition decreased (Figure S2), indicating faster working memory performance, with increasing levels of depression symptoms Table S7C).

### Age-related difference in the working memory advantage

**Figure 4:**
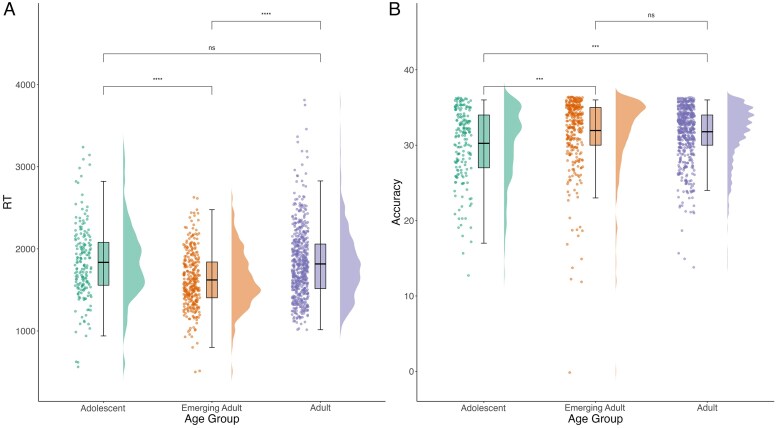
Network task performance depending on age group. *Note*. (A) Participant reaction time in different age groups (adolescent vs. emerging adult vs. adult). Reaction time is operationalized as average reaction time on correct trials of the network task. (B) Participant accuracy in different age groups. Accuracy is operationalized as number of total correct trials out of 36.

Working memory varied across age groups (adolescent vs. emerging adult vs. adult) (RT: *F* = 11.08, *df* = 361.01, *p* < .001; Accuracy: *F* = 5.74, *df* = 361.00, *p* = .004; Table S8). Pairwise comparisons (Table S9) indicated that emerging adults were fastest on the NMT (Figure 4A), and both emerging adults and adults were more accurate compared to adolescents (Figure 4B). However, contrary to our predictions, age was not differentially associated with working memory performance across task conditions or valence (Table S10). In response to reviewer comments, these analyses were additionally run with age entered as a continuous variable. While the observed effect of age (continuous) on RT remained significant, there was no longer a significant effect of age (continuous) on accuracy (*p* > .05; Table S14, Figure S6). The effects of age (continuous) on the remaining preregistered analyses remained consistent with those observed under the different age categories (Table S15). We additionally ran exploratory analyses investigating the effects of age (continuous) on RT using a quadratic mixed-effects model. While there was no significant linear effect of age on RT (*b* = −16.53, *SE* = 8.66, *t* = −1.91, *p* = .057), we observed a significant quadratic effect (*b* = 0.36, *SE* = 0.12, *t* = 2.88, *p* = .004), indicating a U-shaped relationship between age (continuous) and RT.

### Effects of personal networks on working memory

Personal network size, quality, and satisfaction about the opportunities to interact with their social network were unrelated to working memory performance (Table S11).

## Discussion

In this preregistered study, we showed that adolescents demonstrate a working memory advantage for social compared to nonsocial network information, particularly when social information is self-relevant. Adolescents’ recall of positive information in social networks that included themselves was fastest (a self-­positivity bias), whereas negative information was remembered fastest from social networks that included only others (an other-negativity bias).

The observed social working memory advantage aligns with the social brain hypothesis (Dunbar, 1998), which posits that human cognition evolved to compute information on expanding, and increasingly complex social networks. Working memory capacity in our sample improved with age from adolescence to emerging adulthood, with task performance peaking during emerging adulthood. This is consistent with cognitive developmental research (Sawyer et al., 2018), demonstrating that working memory is still developing in adolescence (Blakemore & Choudhury, 2006). However, contrary to our hypotheses, relative to adults, adolescents were not better at processing social compared to nonsocial network information. This may be partially accounted for by unequal groupings, with the adult group including significantly more participants compared to both the adolescent and emerging adult groups. Alternatively, it is possible that this social preference may emerge even earlier in adolescence, between the ages of 10 and 13, to help aid social navigation as individuals first start to become independent from parents.

When dissociating self-relevant from self-irrelevant social information, we observed a self-reference effect (Bentley et al., 2017; Symons & Johnson, 1997) for adolescent network recall. That is, individuals recalled network information fastest and most accurately when it pertained to networks including the self. This self-reference effect may confer additional advantages for successfully navigating complex social networks, as being aware of your social standing could aid with integration into social groups. Indeed, studies have shown that increased awareness of one's own social standing within a group is associated with better relationship management and increased social influence within groups (J. T. Cheng & Tracy, 2014; Galinsky et al., 2008). In adolescence, increased social competence has also been linked to decreased bullying and victimization (Cook et al., 2010). While the findings discussed here indicate that self-relevance may be particularly salient in social contexts, future research should investigate whether this self-reference effect is unique to social environments, or whether a self-reference effect may similarly be observed in nonsocial contexts, by directly comparing self-­relevant working memory performance across social and nonsocial environments. For example, a fourth condition could be introduced to the present task, made up of flight connections between airports that the participants are asked to imagine they are either catching or missing.

Exploratory analyses revealed that individuals performed better in low compared to high cognitive load, recalling network ties more accurately and quickly when networks were made up of connections between 3 rather than 4 individuals or airports. Better working memory performance under low compared to high cognitive load is in line with previous findings that increased cognitive load hinders performance across a range of cognitive functions (Felisberti & Fernandes, 2024; Turcotte & Oddson, 2022).

We also found differential effects of valence across the social conditions. As predicted, adolescents were faster at recalling negative, compared to positive, social information pertaining to others. In contrast, they recalled positive, compared to negative, self-relevant social information faster, contrary to our hypothesis. The self-relevant positivity bias and “other” negativity bias in reaction time for social network information replicate the effects observed in a study conducted with emerging adults and adults (Andrews et al., 2024). Other studies have similarly found a negativity bias for social information across a range of cognition domains, including network memory processing, learning, and decision-making (Fay et al., 2021; Kahneman & Tversky, 1984; Molins et al., 2022). The observed negativity bias could confer advantages to individuals, as awareness of who is disliked within social groups can aid in group integration in novel social environments, which may be critical during adolescence (Chu et al., 2010). Adolescence is also a time when one's self-concept is shaped and is adjusted by social feedback from others (e.g., whether they are liked or disliked; Crone et al., 2022). Observing a positivity bias for self-relevant social information, then, is promising, as it may contribute toward building more positive self-concepts and self-esteem (Alsaker & Kroger, 2007), which in turn are associated with greater wellbeing across the lifespan (Orth & Robins, 2022). It should be noted that these self-positivity and other-negativity effects were not observed for accuracy. Reaction time has been shown to be more sensitive than accuracy to moderating effects of social-affective salience (Schweizer et al., 2019).

Contrary to our hypothesis, social sensitivity did not potentiate the social working memory advantage or self-relevance effect. This may be in part due to overall high levels of social sensitivity reported in the current sample, consistent with research findings that social sensitivity is heightened in adolescence (Somerville, 2013). However, age-related variance in social sensitivity did not moderate the effect of sociality (social vs. nonsocial networks) on working memory performance, suggesting instead that social sensitivity may not interact with the effect of fictional networks and instead only plays out in real-world networks. Interestingly, however, depressive symptoms, for which social sensitivity is a risk factor, did modulate the social advantage effect. That is, higher levels of depressive symptoms were associated with faster correct responses on the NMT in the social relative to the nonsocial condition when controlling for valence. This finding is noteworthy, as depressive symptoms are generally associated with impaired cognition across executive functions, including working memory (Snyder, 2013). Together, these findings suggest that despite widespread executive functioning deficits observed in individuals with high levels of depressive symptoms, social information processing, maintenance, and recall appear relatively preserved and are arguably prioritized. This may be accounted for by the hypothesized sensitization to social affiliative information in individuals with high levels of depressive symptoms (Allen & Badcock, 2003). It may also promote social functioning and thereby facilitate social support, which has been shown to be protective in individuals with high levels of depressive symptoms.

The association between social working memory capacity and social functioning, however, remains speculative, as social working memory performance was unrelated to real-world network characteristics, such as size and quality, in the current sample. Better performance in social situations (e.g., knowing who to approach or avoid in social contexts) may lead to establishing larger social networks over time. Future research could then examine these proposed effects across development, as these differences in network formation may not yet be observable during adolescence, becoming more evident as social network compositions stabilize before decreasing again in later adulthood (Wrzus et al., 2013). Working memory for social networks may also be differentially associated with family versus friendship networks, which were not dissociated in the current study. More fine-grained understanding of the association between social working memory and individuals’ social network compositions are therefore needed.

Interestingly, the size of real-world networks reported by participants in the present study closely aligned with theoretical models of network size and formation. It has been hypothesized that there are limits to the size of individuals’ social networks (Dunbar, 1993). These estimations suggest that humans typically have approximately 5 close relations, and the number of relations increases as the level of closeness decreases (Zhou et al., 2005). In line with this, in the present study participants reported having on average approximately 4 close friends in total that they would trust enough to do a favor or keep a secret. Similarly, participants reported having on average approximately 550 “friends” on social media, which also closely aligns with the proposed 500 people an individual can maintain as acquaintances (Zhou et al., 2005).

In conclusion, this study presents evidence for a human social working memory advantage across adolescence and adulthood, especially when the information is self-relevant. We also show that individuals display a positivity bias for recall of self-relevant social information, and a negativity bias for social information pertaining only to “others”. Lastly, findings suggest that working memory abilities are differentially affected by age, improving during emerging adulthood. As the sample was relatively diverse, findings should have moderate generalizability. These insights contribute to our understanding of how social information is processed in memory systems, and the factors that facilitate the successful navigation of the complex social environments that make up human society.

## Supplementary Material

aacaf039_Supplementary_Data

## Data Availability

The analyses presented in this study were preregistered prior to data collection. The preregistration is available at the following URL: https://osf.io/brnxc. De-identified data and code necessary to reproduce the analyses presented are accessible at https://osf.io/brnx. The materials necessary to attempt to replicate the findings presented here are not publicly available, as some of the included images are licensed to the authors.
