# Supplementary material for: Social working memory in adolescence
Source: Child Dev. 2026 Jan 29;97(2):482–90. doi: 10.1093/chidev/aacaf039 (PMC13046068; doi:10.1093/chidev/aacaf039)
Supplement: aacaf039_Supplementary_Data [file aacaf039_supplementary_data.docx]

**Network Study Follow-up**

**Supplementary Materials**

**Excluded Participants**

Two of the recruited participants did not meet the attention check requirements, and four were flagged as likely to be duplicate or fraudulent participants when completing the initial Qualtrics questionnaire and then proceeded to score below chance on the NMT. These six participants were excluded and another six were recruited in their place. Additionally, after completing the recruitment target for emerging adults, adolescents were still being recruited, and one of the individuals that initially reported being 13-17 through Qualtrics then indicated that they were part of the emerging adults age group when completing the experiment demographics. They therefore had to be added to the emerging adults sample and an additional adolescent participant was recruited in their place. Altogether, this led to a total recruitment of 135 participants (64 aged 13-17 years; 65 aged 18-24 years) between January and June 2023. After the initial 6 participants mentioned above were excluded after the recruitment phase, a further 6 participants were excluded from data analyses for performing below chance on the network task (responding accurately on less than 50% of the trials). The final sample comprised 123 participants.

**Final Sample and Model Overview**

Participants (*N* = 123) recruited through Prolific academic, MQ participate, and Facebook advertising were between 13-24 years (*n* = 60 13-17 years; *n* = 63 18-24 years). Participant characteristics are reported in Table S1A. For hypothesis 3 analyses, this sample was combined with a sample from a previous study conducted with emerging adults and adults (18-65 years; Andrews et al., 2024). The full sample for these analyses was *N* = 364 of ages between 13-65 years (*n* = 60 13-17 years; *n* = 111 18-24 years; *n* = 193 25-65 years). Participant characteristics for these analyses are reported in Table S1B.

**Table S1**

*Sample Characteristics (N = 123), Means and Standard Deviations of Variables*

|  | ***A: adolescent sample***  ***(N = 123)*** | ***B: combined samples***  ***(N = 364)*** |
| --- | --- | --- |
|  | ***N (%)*** | ***N (%)*** |
| **Gender** |  |  |
| Female | 65 (52.85) | 208 (57.14) |
| Male | 57 (46.34) | 153 (42.03) |
| Non-Binary | 0 (0) | 2 (0.55) |
| Prefer not to say | 1 (0.81) | 1 (0.27) |
| **Ethnicity** |  |  |
| Aboriginal/Torres Strait  Islander | 0 (0) | 3 (0.82) |
| Black | 4 (3.25) | 19 (5.22) |
| Asian | 23 (18.70) | 63 (17.31) |
| White | 84 (68.29) | 240 (65.93) |
| Hispanic | 1 (0.81) | 8 (2.20) |
| Mixed | 7 (5.69) | 18 (4.95) |
| Prefer not to say | 2 (1.63) | 5 (1.37) |
| Other | 2 (1.63) | 8 (2.20) |
| **Highest Education** |  |  |
| Primary School | 3 (2.44) | 3 (0.82) |
| High School | 79 (64.23) | 136 (37.36) |
| Training | 5 (4.06) | 38 (10.44) |
| University | 36 (29.27) | 187 (51.37) |
| **Wealth** |  |  |
| Not at all | 1 (0.81) | 24 (6.59) |
| Not very | 25 (20.33) | 110 (30.22) |
| Fairly | 63 (51.22) | 171 (46.98) |
| Rather | 31 (25.20) | 55 (15.11) |
| Very | 3 (2.44) | 4 (1.10) |
| **Most used SM platform** |  |  |
| Discord | 10 (8.13) | N/A |
| Facebook | 27 (21.95) | N/A |
| Instagram | 54 (43.90) | N/A |
| Snapchat | 21 (17.07) | N/A |
| Twitter | 5 (4.07) | N/A |
| Other | 6 (4.88) | N/A |
|  | ***Mean (SD)*** | ***Mean (SD)*** |
| **Age (years)** | 18.54 (3.16) | 29.02 (11.75) |
| **Depressive symptoms** | 12.15 (11.27) | 12.28 (10.44) |
| **Social sensitivity** | 25.49 (10.08) | 23.84 (10.67) |
| **Size** | 14.18 (12.36) | N/A |
| In person | 5.85 (4.93) | N/A |
| Online | 8.33 (9.31) | N/A |
| **Quality** | 8.30 (9.26) | N/A |
| Favour | 4.56 (5.99) | N/A |
| Secret | 3.74 (3.94) | N/A |
| **Satisfaction** | 141.61 (44.93) | N/A |
| In person | 70 (27.41) | N/A |
| Online | 71.61 (23.23) | N/A |
| **Cool ratio** | 1.76 (4.76) | N/A |
| People they follow | 461.80 (693.07) | N/A |
| People that follow them | 633.63 (987.87) | N/A |
| **RT social-self** | 1651.38 (393.29) | 1700.50 (396.00) |
| Positive Valence | 1582.00 (394.26) | 1636.08 (402.27) |
| Negative Valence | 1724.20 (414.53) | 1765.53 (413.93) |
| **RT social-other** | 1737.29 (403.75) | 1757.89 (414.88) |
| Positive Valence | 1781.55 (442.77) | 1807.22 (439.53) |
| Negative Valence | 1693.29 (425.31) | 1707.79 (435.49) |
| **RT non-social** | 1768.64 (439.85) | 1823.27 (427.74) |
| Positive Valence | 1793.75 (446.47) | 1845.43 (454.98) |
| Negative Valence | 1746.33 (465.21) | 1800.13 (440.90) |
| **Accuracy social-self** | 31.90 (4.76) | 32.46 (3.82) |
| Positive Valence | 16.15 (2.50) | 16.40 (2.02) |
| Negative Valence | 15.76 (2.65) | 16.08 (2.16) |
| **Accuracy social-other** | 30.98 (4.36) | 31.42 (3.87) |
| Positive Valence | 15.66 (2.33) | 15.73 (2.12) |
| Negative Valence | 15.32 (2.53) | 15.69 (2.17) |
| **Accuracy non-social** | 30.30 (5.00) | 30.84 (4.58) |
| Positive Valence | 15.22 (2.85) | 15.43 (2.47) |
| Negative Valence | 15.08 (2.83) | 15.41 (2.60) |

*Note*. Training = Professional/Vocational Training. Wealth = how wealthy participants think they are. Depressive symptoms = total score on the short-version of the Depression, Anxiety and Stress Scale (DASS-21; Lovibond & Lovibond, 1995) depression subscale; Social sensitivity = total score on the Online and Offline Social Sensitivity Scale (O^2^S^3^; Andrews et al., 2022); Size = sum of total number of in person and online friends; Quality = sum of total number of friends participants could ask a favour of and would trust to keep a secret; Satisfaction = sum of reported happiness with how often participants spend time with friends online and in person (on a scale from 1-200); Cool ratio = ratio of the number of people participants follow on social media compared to the number of people that follow participants on social media; RT social-self = reaction time for social-self network task condition; RT social-other = reaction time for social-other network task condition; RT non-social = reaction time for non-social network task condition; Accuracy social-self = accuracy for social-self network task condition; Accuracy social-other = accuracy for social-other network task condition; Accuracy non-social = accuracy for non-social network task condition; Positive Valence = network associations where people were friends/flights were running; Negative Valence = network associations where people were not friends/flights were not running.

**Figure S1**

*Study Procedure Flow Chart*

*
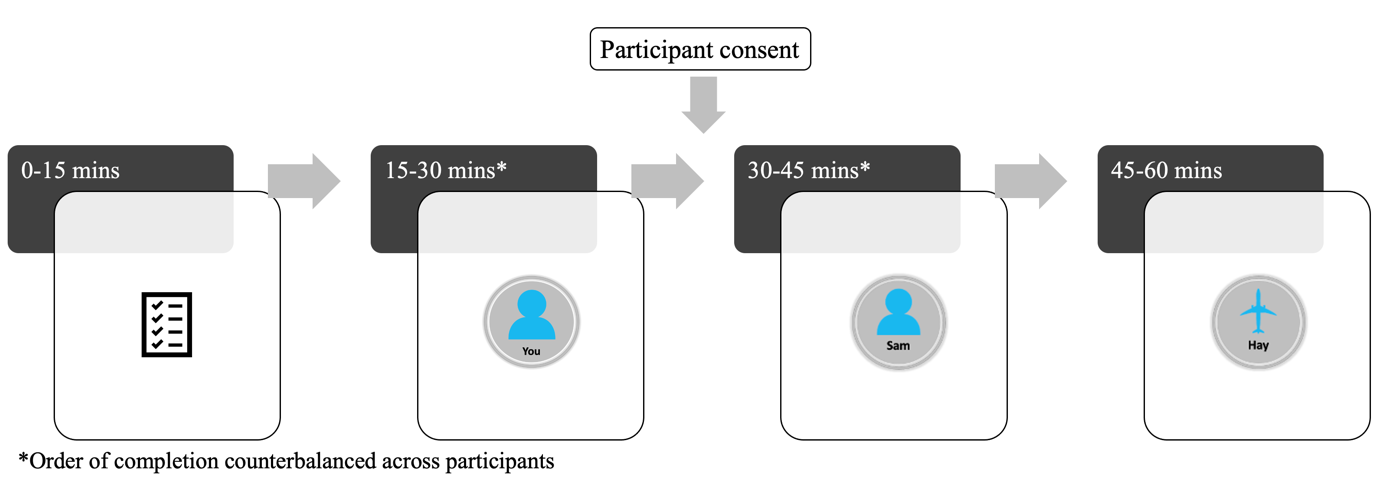
*

*Note*. Study procedure. **
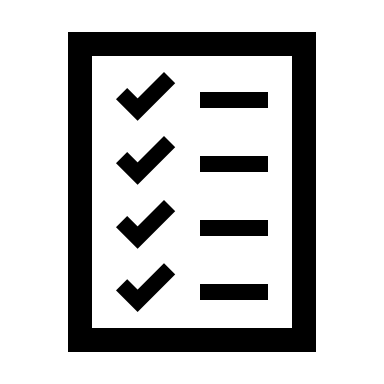
** = self-report measures (participant information sheet and consent, demographics questionnaire, DASS-21, O^2^S^3^, real-world social networks); **
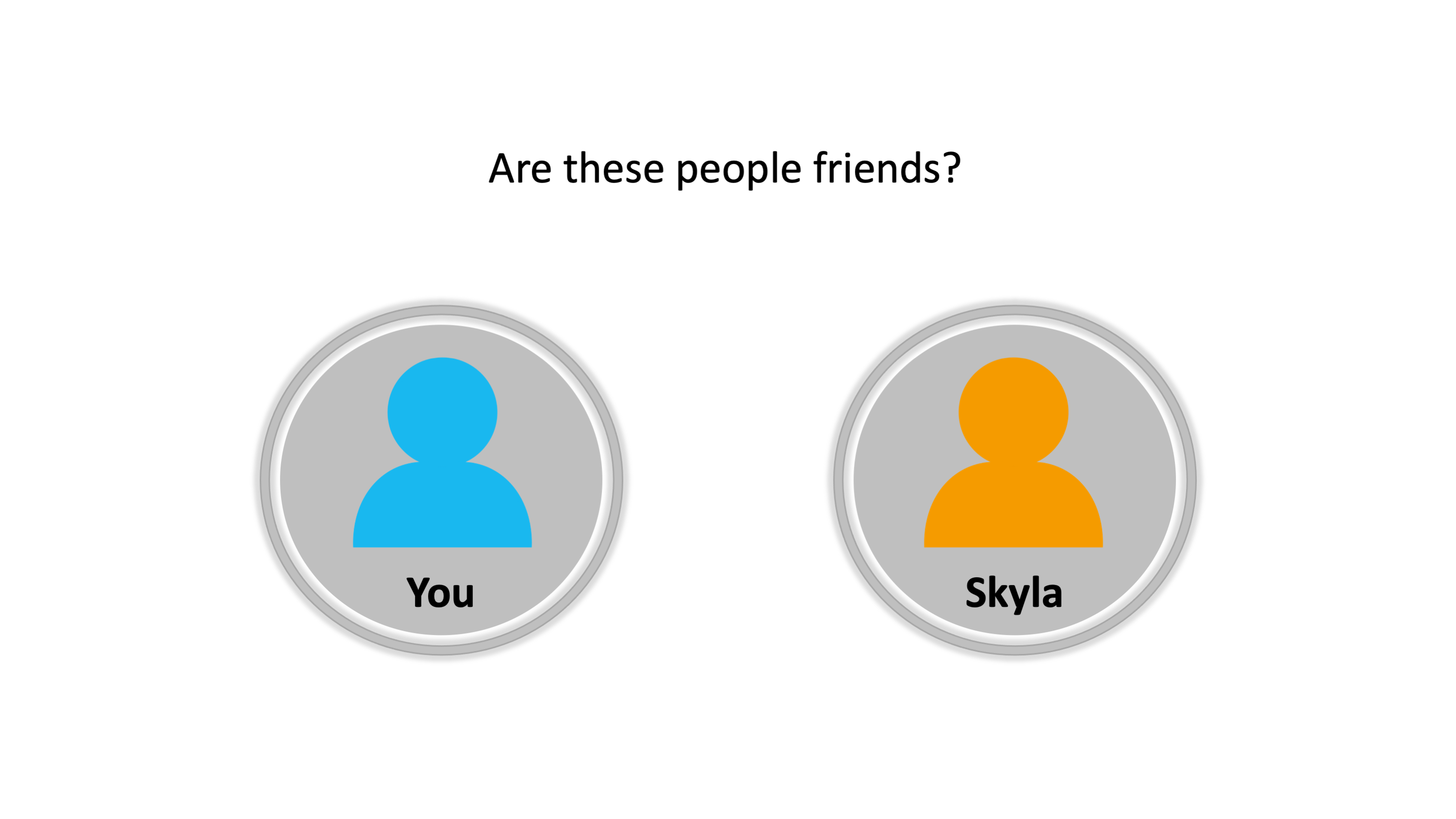
**= network memory task (social-self condition); **
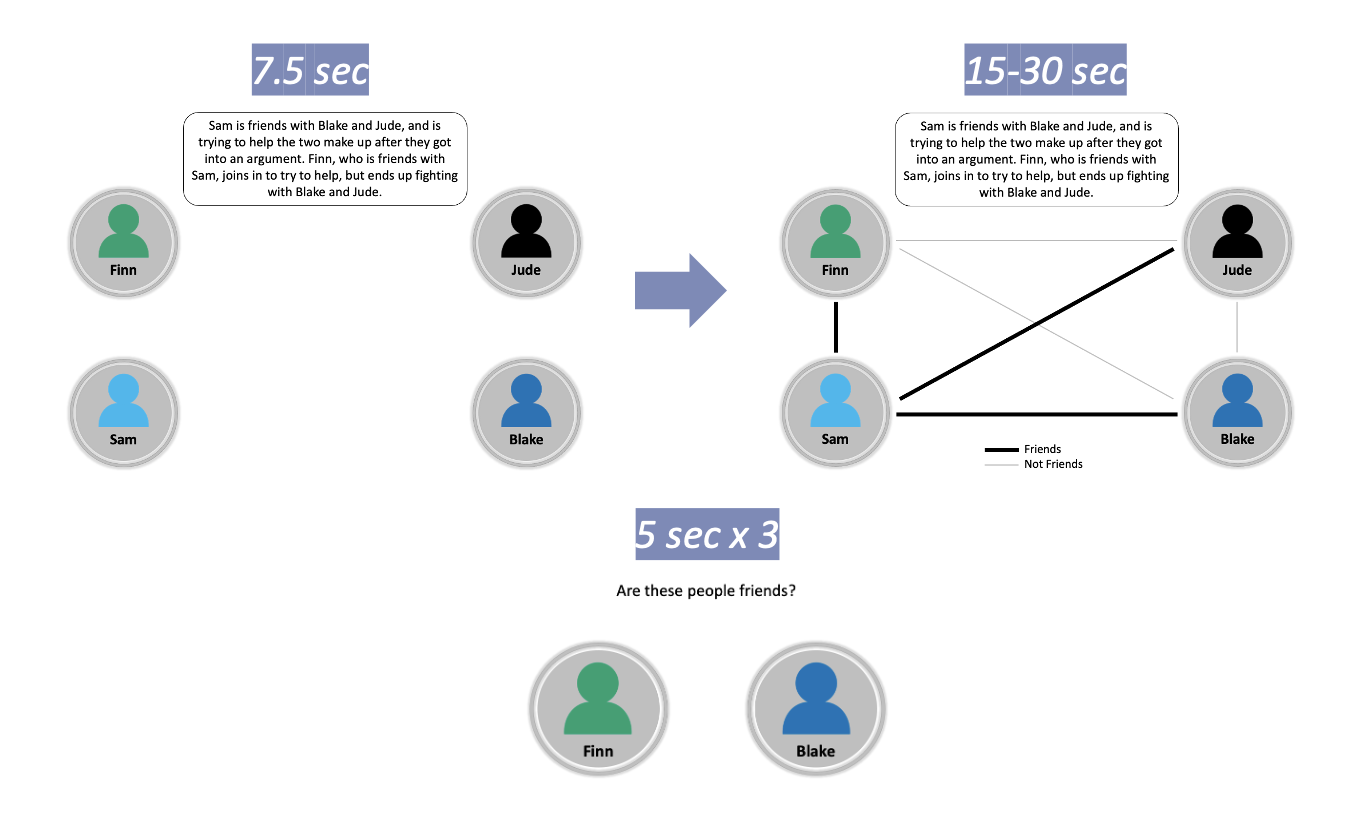
**= network memory task (social-other condition); **
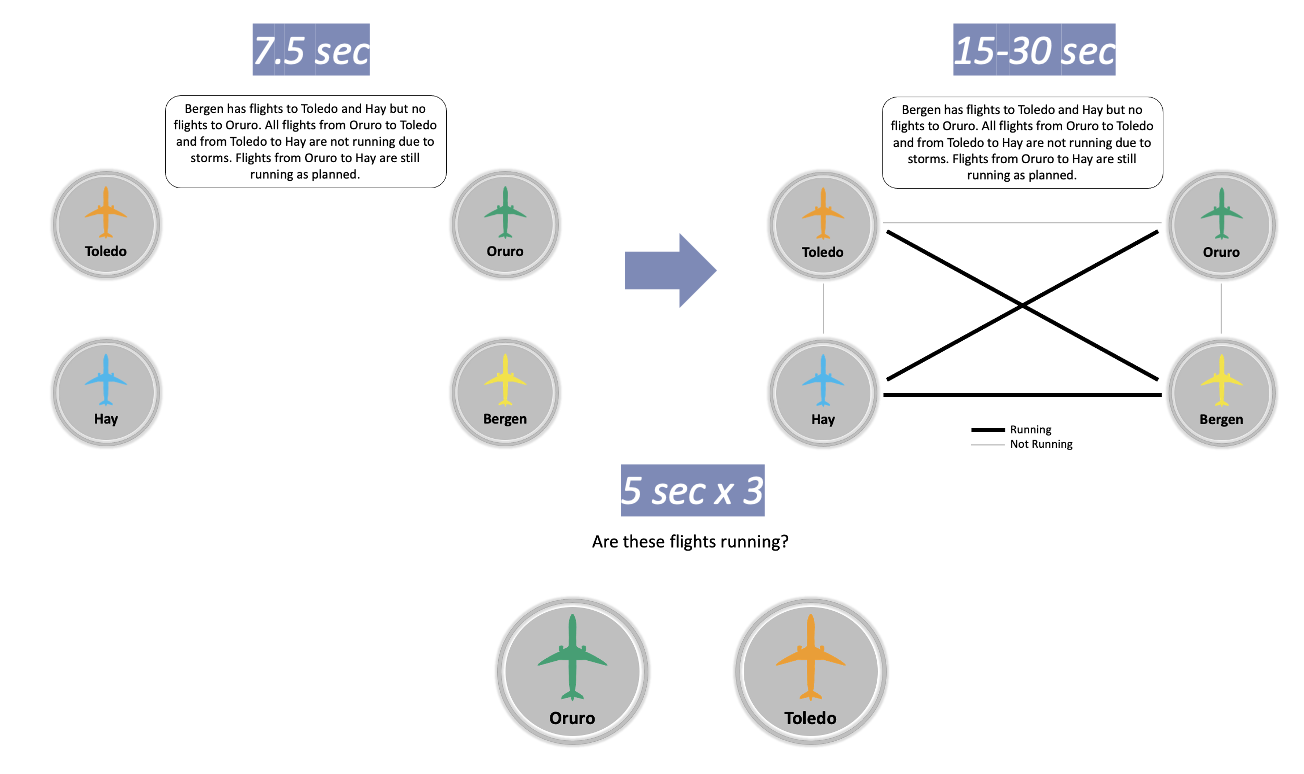
**= network memory task (non-social condition).

**R Packages**

Analyses were conducted including the following R packages. Linear mixed models were conducted using the lme4 package (Bates et al., 2015). Significant effects were further investigated with contrasts using the lsmeans package (Lenth, 2017). Correlations were conducted using the apaTables package (Stanley & Stanley, 2018). Effect sizes were calculated using the MuMIn package (Bartoń, 2010).

**Table S2**

*Correlations of Predictor and Outcome Variables*

| Variable | 1 | 2 | 3 | 4 | 5 | 6 | 7 | 8 | 9 | 10 | 11 | 12 |
| --- | --- | --- | --- | --- | --- | --- | --- | --- | --- | --- | --- | --- |
| 1. Age |  |  |  |  |  |  |  |  |  |  |  |  |
| 2. Depressive symptoms | .10 |  |  |  |  |  |  |  |  |  |  |  |
| 3. Social sensitivity | -.03 | **.61** |  |  |  |  |  |  |  |  |  |  |
| 4. Size | -.14 | **-.24** | **-.21** |  |  |  |  |  |  |  |  |  |
| 5. Quality | **.20** | -.16 | **-.27** | **.58** |  |  |  |  |  |  |  |  |
| 6. Satisfaction | **-.34** | **-.44** | **-.29** | .15 | .03 |  |  |  |  |  |  |  |
| 7. Cool ratio | .01 | .00 | -.01 | -.02 | -.05 | .17 |  |  |  |  |  |  |
| 8. RT self | **-.27** | -.11 | -.04 | .14 | -.12 | .08 | -.11 |  |  |  |  |  |
| 9. RT other | **-.25** | -.18 | -.04 | .10 | -.08 | .11 | -.17 | **.81** |  |  |  |  |
| 10. RT non | **-.20** | -.05 | -.02 | .08 | -.06 | .03 | -.14 | **.81** | **.81** |  |  |  |
| 11. Accuracy self | .13 | .11 | -.01 | .04 | .06 | **-.19** | -.14 | -.01 | -.10 | .07 |  |  |
| 12. Accuracy other | **.22** | -.01 | -.04 | .06 | .17 | -.13 | **-.25** | .06 | -.00 | .11 | **.55** |  |
| 13. Accuracy non | **.20** | .01 | -.06 | -.07 | .10 | **-.18** | **-.32** | -.05 | -.11 | .02 | **.53** | **.71** |

*Note*. Bolded text indicates statistically significant effects. Depressive symptoms = total score on the DASS-21 depression subscale; Social sensitivity = total score on the O^2^S^3^; Size = sum of total number of in person and online friends; Quality = sum of total number of friends participants could ask a favour of and would trust to keep a secret; Satisfaction = sum of reported happiness with how often participants spend time with friends online and in person; Cool ratio = ratio of the number of people participants follow on social media compared to the number of people that follow participants on social media; RT self = reaction time for social-self network task condition; RT other = reaction time for social-other network task condition; RT non = reaction time for non-social network task condition; Accuracy self = accuracy for social-self network task condition; Accuracy other = accuracy for social-other network task condition; Accuracy non = accuracy for non-social network task condition.

**Supplementary Results**

**Table S3**

*Summary of H1 Analyses*

|  | **Reaction Time** | | | | **Accuracy** | | | |
| --- | --- | --- | --- | --- | --- | --- | --- | --- |
| *Predictors* | *df* | *F* | *p* | *R^2^m/R^2^c* | *df* | *F* | *p* | *R^2^m/R^2^c* |
| ***A:*** *H1a Analyses* | | | | | | | | |
|  |  |  |  | 0.01/0.80 |  |  |  | 0.01/0.59 |
| Condition | **245.00** | **13.04** | **<.001** |  | **245.00** | **11.55** | **.001** |  |
| ***B:*** *H1b Analyses* | | | | | | | |  |
|  |  |  |  | 0.01/0.81 |  |  |  | 0.02/0.60 |
| Condition | **244.00** | **13.74** | **<.001** |  | **244.00** | **8.82** | **<.001** |  |
| ***C:*** *H1c Analyses (Social vs Non-Social Condition)* | | | | | | | | |
|  |  |  |  | 0.01/0.73 |  |  |  | 0.01/0.51 |
| Condition | 611.09 | 1.99 | .159 |  | **612.00** | **4.96** | **.026** |  |
| Valence | 611.07 | 2.92 | .088 |  | 612.00 | 0.34 | .558 |  |
| Condition x Valence | **611.05** | **4.66** | **.031** |  | 612.00 | 0.62 | .431 |  |
| ***D:*** *H1c Analyses (Social-Self, Social-Other vs Non-Social Condition)* | | | | | | | | |
|  |  |  |  | 0.03/0.75 |  |  |  | 0.02/0.52 |
| Condition | 609.04 | 1.70 | .184 |  | **610.00** | **4.25** | **.015** |  |
| Valence | 609.06 | 3.17 | .076 |  | 610.00 | 0.35 | .557 |  |
| Condition x Valence | 609.02 | 19.74 | **<.001** |  | 610.00 | 0.32 | .724 |  |

*Note*. Bolded text indicates statistically significant effects. Analyses were conducted on the adolescent sample. H1 investigated the effects of condition (H1a: social vs non-social; H1b: social-self vs social-other vs non-social) on task performance (i.e., RT and accuracy), and whether these effects would differ as a function of valence (H1c).

**Table S4**

*Summary of Contrast Models for H1b (3-level Condition) Analyses*

|  | **Reaction Time** | | | | **Accuracy** | | | |
| --- | --- | --- | --- | --- | --- | --- | --- | --- |
| *Predictors* | *β* | *95% CI* | *t* | *p* | *β* | *95% CI* | *t* | *p* |
| Non vs Other | 0.08 | −0.06 - 0.21 | 1.35 | .443 | −0.14 | −0.34 - 0.05 | − 1.76 | .220 |
| Non vs Self | **0.28** | **0.15 - 0.42** | **5.06** | **<.001** | **−0.34** | **−0.53 - −0.14** | **−4.18** | **<.001** |
| Other vs Self | **0.21** | **0.07 - 0.34** | **3.71** | **.001** | **−0.20** | **−0.39 - −0.00** | **−2.42** | **.048** |

*Note*. Bolded text indicates statistically significant effects. Non indicates non-social condition; Other indicates social-other condition; Self indicates social-self condition. Analyses were conducted on the adolescent sample. H1b investigated the effects of condition (social-self vs social-other vs non-social) on task performance (i.e., RT and accuracy).

**Table S5**

*Summary of Contrast Models for H1c Analyses*

|  | **Reaction Time** | | | | **Accuracy** | | | |
| --- | --- | --- | --- | --- | --- | --- | --- | --- |
| *Predictors* | *β* | *95% CI* | *t* | *p* | *β* | *95% CI* | *t* | *p* |
| ***A:*** *Social vs Non-Social (Condition)* | | | | | | | | |
| Non (Negative vs Positive) | −0.11 | −0.28 - 0.05 | −1.71 | .307 | −0.05 | −0.28 - 0.17 | −0.59 | .962 |
| Social (Negative vs Positive) | 0.06 | −0.06 - 0.18 | 1.32 | .564 | −0.14 | −0.30 - 0.02 | −2.19 | .110 |
| Non vs Social (Negative) | 0.08 | −0.06 - 0.23 | 1.41 | .499 | −0.17 | −0.37 - 0.02 | −2.23 | .101 |
| Non vs Social (Positive) | **0.26** | **0.11 - 0.40** | **4.47** | **<.001** | −0.26 | −0.45 - −0.07 | −3.34 | .004 |
| ***B:*** *Social-Self, Social-Other vs Non-Social (Condition)* | | | | | | | | |
| Non (Negative vs Positive) | −0.11 | −0.29 - 0.06 | −1.78 | .507 | −0.05 | −0.30 - 0.19 | −0.59 | .999 |
| Other (Negative vs Positive) | **−0.20** | **−0.38 - −0.03** | **−3.18** | **.014** | −0.13 | −0.38 - 0.12 | −1.45 | .760 |
| Self (Negative vs Positive) | **0.33** | **0.15 - 0.50** | **5.12** | **<.001** | −0.15 | −0.40 - 0.10 | −1.66 | .602 |
| Non vs Other (Negative) | 0.12 | −0.06 - 0.29 | 1.83 | .470 | −0.09 | −0.34 - 0.16 | −1.00 | .967 |
| Non vs Self (Negative) | 0.05 | −0.13 - 0.22 | 0.72 | .997 | −0.26 | −0.50 - −0.01 | −2.87 | .037 |
| Other vs Self (Negative) | −0.07 | −0.25 - 0.11 | −1.11 | .938 | −0.17 | −0.41 - 0.08 | −1.87 | .439 |
| Non vs Other (Positive) | 0.03 | −0.15 - 0.20 | 0.44 | .999 | −0.17 | −0.41 - 0.08 | −1.87 | .439 |
| Non vs Self (Positive) | **0.49** | **0.31 - 0.66** | **7.62** | **<.001** | −0.35 | −0.60 - −0.10 | −3.95 | .001 |
| Other vs Self (Positive) | **0.46** | **0.28 - 0.63** | **7.19** | **<.001** | −0.18 | −0.43 - 0.06 | −2.08 | .296 |

*Note*. Bolded text indicates statistically significant effects. Non indicates non-social condition; Other indicates social-other condition; Self indicates social-self condition. Analyses were conducted on the adolescent sample. H1c investigated whether the effects of condition (social vs non-social; social-self vs social-other vs non-social) on task performance (i.e., RT and accuracy) would differ as a function of valence.

**Table S6**

*Summary of H2 Analyses*

|  | **Reaction Time** | | | | **Accuracy** | | | |
| --- | --- | --- | --- | --- | --- | --- | --- | --- |
| *Predictors* | *df* | *F* | *p* | *R^2^m/R^2^c* | *df* | *F* | *p* | *R^2^m/R^2^c* |
| ***A:*** *H2a Analyses* | | | | | | | | |
|  |  |  |  | 0.01/0.80 |  |  |  | 0.01/0.59 |
| Condition | 244.00 | 1.00 | .319 |  | 244.00 | 0.59 | .444 |  |
| Social sensitivity | 159.63 | 0.07 | .792 |  | 214.52 | 0.44 | .506 |  |
| Condition x Social sensitivity | 244.00 | 0.12 | .729 |  | 244.00 | 0.26 | .609 |  |
| ***B:*** *H2b Analyses* | | | | | | | | |
|  |  |  |  | 0.02/0.81 |  |  |  | 0.02/0.60 |
| Condition | 242.00 | 1.38 | .254 |  | 242.00 | 0.44 | .644 |  |
| Social sensitivity | 157.83 | 0.07 | .792 |  | 212.98 | 0.45 | .505 |  |
| Condition x Social sensitivity | 242.00 | 0.06 | .938 |  | 242.00 | 0.20 | .816 |  |
| ***C:*** *H2c Analyses (Social vs Non-Social Condition)* | | | | | | | | |
|  |  |  |  | 0.01/0.73 |  |  |  | 0.02/0.52 |
| Condition | 608.21 | 0.08 | .784 |  | 609.00 | 0.97 | .324 |  |
| Valence | 608.16 | 0.02 | .898 |  | **609.00** | **6.09** | **.014** |  |
| Social sensitivity | 199.16 | 0.27 | .607 |  | 316.75 | 0.36 | .549 |  |
| Condition x Valence | 608.11 | 2.17 | .141 |  | 609.00 | 0.25 | .616 |  |
| Condition x Social sensitivity | 608.14 | 0.72 | .395 |  | 609.00 | 0.03 | .858 |  |
| Valence x Social sensitivity | 608.11 | 0.29 | .593 |  | **609.00** | **5.86** | **.016** |  |
| Condition x Valence x Social sensitivity | 608.07 | 0.54 | .462 |  | 609.00 | 0.72 | .395 |  |
| ***D:*** *H2c Analyses (Social-Self, Social-Other vs Non-Social Condition)* | | | | | | | | |
|  |  |  |  | 0.03/0.75 |  |  |  | 0.03/0.52 |
| Condition | 604.10 | 0.04 | .957 |  | 605.00 | 0.51 | .601 |  |
| Valence | 604.14 | 0.02 | .893 |  | **605.00** | **6.14** | **.013** |  |
| Social sensitivity | 192.91 | 0.27 | .604 |  | 314.94 | 0.36 | .549 |  |
| Condition x Valence | 604.05 | 2.64 | .072 |  | 605.00 | 0.53 | .591 |  |
| Condition x Social sensitivity | 604.06 | 0.45 | .636 |  | 605.00 | 0.46 | .630 |  |
| Valence x Social sensitivity | 604.10 | 0.31 | .579 |  | **605.00** | **5.91** | **.015** |  |
| Condition x Valence x Social sensitivity | 604.03 | 0.40 | .669 |  | 605.00 | 0.77 | .463 |  |

*Note*. Bolded text indicates statistically significant effects. Analyses were conducted on the adolescent sample. H2 investigated the effects of condition (H2a: social vs non-social; H2b: social-self vs social-other vs non-social) and social sensitivity on task performance (i.e., RT and accuracy), and whether these effects would differ as a function of valence (H2c).

**Table S7**

*Exploring Effects of Depressive Symptoms on H2 Analyses*

|  | **Reaction Time** | | | | **Accuracy** | | | |
| --- | --- | --- | --- | --- | --- | --- | --- | --- |
| *Predictors* | *df* | *F* | *p* | *R^2^m/R^2^c* | *df* | *F* | *p* | *R^2^m/R^2^c* |
| ***A:*** *H2a Analyses* | | | | | | | | |
|  |  |  |  | 0.02/0.80 |  |  |  | 0.01/0.59 |
| Condition | 244.00 | 1.22 | .270 |  | 244.00 | 3.49 | .063 |  |
| Depressive symptoms | 159.60 | 0.29 | .593 |  | 214.48 | 0.01 | .932 |  |
| Condition x Depressive symptoms | 244.00 | 3.41 | .066 |  | 244.00 | 0.35 | .555 |  |
| ***B:*** *H2b Analyses* | | | | | | | | |
|  |  |  |  | 0.03/0.81 |  |  |  | 0.02/0.61 |
| Condition | **242.0** | **6.28** | **.002** |  | 242.00 | 1.93 | .147 |  |
| Depressive symptoms | 157.58 | 0.29 | .591 |  | 212.17 | 0.01 | .932 |  |
| Condition x Depressive symptoms | 242.0 | 2.43 | .090 |  | 242.00 | 1.33 | .268 |  |
| ***C:*** *H2c Analyses (Social vs Non-Social Condition)* | | | | | | | | |
|  |  |  |  | 0.02/0.74 |  |  |  | 0.02/0.52 |
| Condition | 608.16 | 0.47 | .493 |  | 609.00 | 2.54 | .112 |  |
| Valence | 608.12 | 0.88 | .350 |  | **609.00** | **4.85** | **.028** |  |
| Depressive symptoms | 198.82 | 0.46 | .500 |  | 316.54 | 1.67 | .197 |  |
| Condition x Valence | **608.08** | **5.03** | **.025** |  | 609.00 | 0.00 | .944 |  |
| Condition x Depressive symptoms | **608.07** | **5.04** | **.025** |  | 609.00 | 0.01 | .923 |  |
| Valence x Depressive symptoms | 608.06 | 0.10 | .751 |  | **609.00** | **6.02** | **.014** |  |
| Condition x Valence x Depressive symptoms | 608.04 | 1.10 | .295 |  | 609.00 | 0.68 | .409 |  |
| ***D:*** *H2c Analyses (Social-Self vs Social-Other vs Non-Social Condition)* | | | | | | | | |
|  |  |  |  | 0.04/0.76 |  |  |  | 0.03/0.53 |
| Condition | 604.07 | 0.28 | .759 |  | 605.00 | 1.30 | .273 |  |
| Valence | 604.11 | 0.95 | .330 |  | **605.00** | **4.91** | **.027** |  |
| Depressive symptoms | 192.30 | 0.47 | .496 |  | 314.00 | 1.68 | .196 |  |
| Condition x Valence | **604.04** | **9.44** | **<.001** |  | 605.00 | 0.06 | .937 |  |
| Condition x Depressive symptoms | **604.03** | **3.59** | **.028** |  | 605.00 | 1.11 | .331 |  |
| Valence x Depressive symptoms | 604.05 | 0.11 | .741 |  | **605.00** | **6.09** | **.014** |  |
| Condition x Valence x Depressive symptoms | 604.02 | 0.71 | .494 |  | 605.00 | 0.41 | .667 |  |

*Note*. Bolded text indicates statistically significant effects. Analyses were conducted on the adolescent sample. These analyses investigated the effects of condition (H2a: social vs non-social; H2b: social-self vs social-other vs non-social) and depressive symptoms on task performance (i.e., RT and accuracy), and whether these effects would differ as a function of valence (H2c).

**Figure S2**

*Network Task Performance Depending on Depressive Symptoms and Condition*


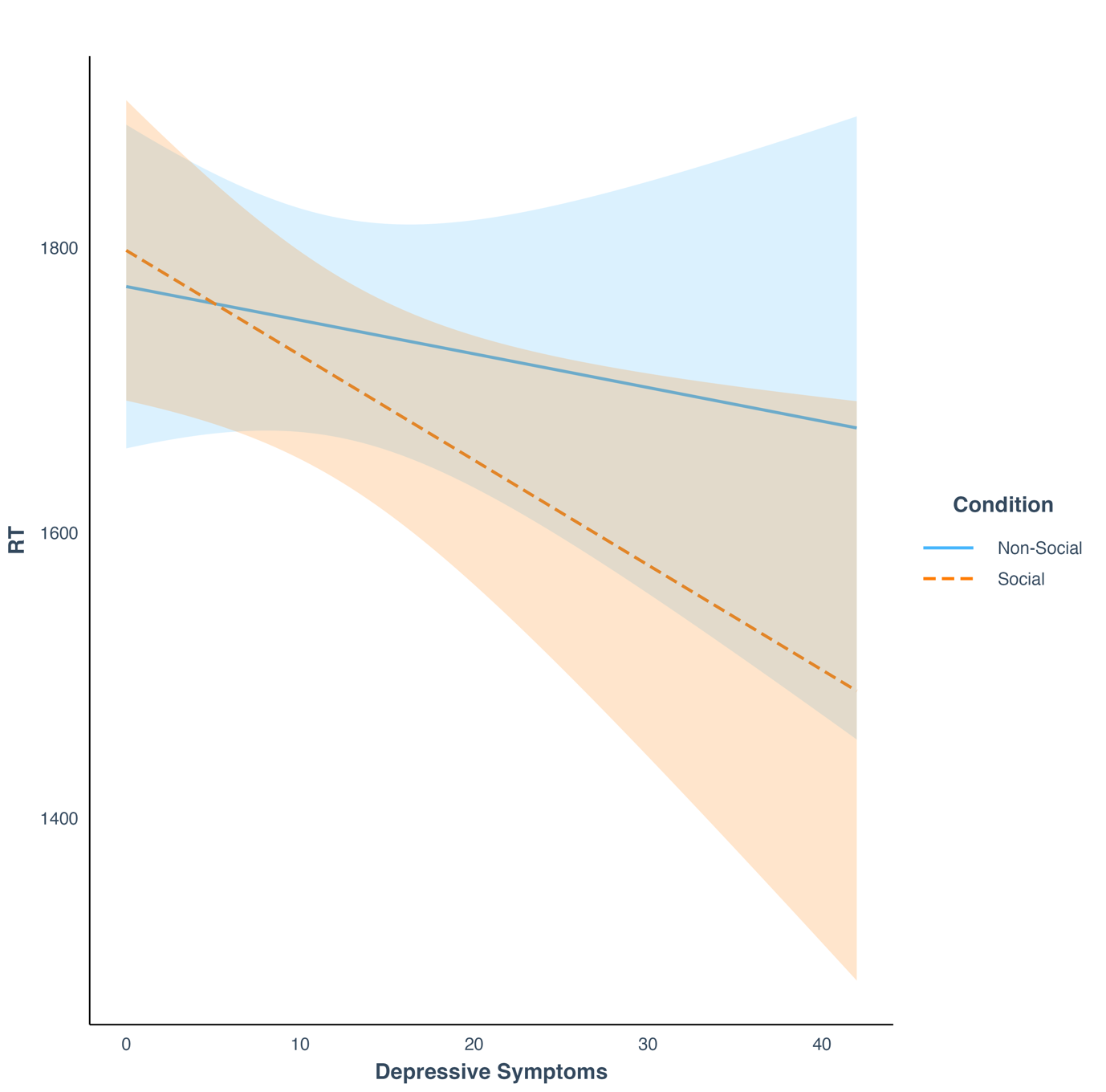


*Note*. Interaction effects between depressive symptoms (total score on the short-version of the DASS-21) and condition (non-social vs social) on participant reaction time (RT) in the network task. Reaction time is operationalised as average reaction time on correct trials of the network task.

**Table S8**

*Summary of Preliminary Age Analyses*

|  | **Reaction Time** | | | | **Accuracy** | | | |
| --- | --- | --- | --- | --- | --- | --- | --- | --- |
| *Predictors* | *df* | *F* | *p* | *R^2^m/R^2^c* | *df* | *F* | *p* | *R^2^m/R^2^c* |
|  |  |  |  | 0.05/0.78 |  |  |  | 0.02/0.50 |
| Age | **361.01** | **11.08** | **<.001** |  | **361.00** | **5.74** | **.004** |  |

*Note*. Bolded text indicates statistically significant effects. Age = adolescent vs adult vs emerging adult. Analyses were conducted on the combined sample. These analyses investigated the effects of age (adolescent vs emerging adult vs adult) on task performance (i.e., RT and accuracy).

**Table S9**

*Summary of Contrast Models for Preliminary Age Analyses*

|  | **Reaction Time** | | | | **Accuracy** | | | |
| --- | --- | --- | --- | --- | --- | --- | --- | --- |
| *Predictors* | *β* | *95% CI* | *t* | *p* | *β* | *95% CI* | *t* | *p* |
| Adolescent vs Adult | 0.05 | −0.27 - 0.36 | 0.34 | .981 | **−0.37** | **−0.66 - −0.08** | **−3.10** | **.006** |
| Adolescent vs Emerging | **0.52** | **0.17 - 0.86** | **3.58** | **.001** | **−0.41** | **−0.72 - −0.10** | **−3.16** | **.005** |
| Adult vs Emerging | **0.47** | **0.21 - 0.73** | **4.39** | **<.001** | −0.04 | −0.27 - 0.19 | −0.40 | .970 |

*Note*. Bolded text indicates statistically significant effects. Adolescent indicates adolescent age group; Emerging indicates emerging adult age group; Adult indicates adult age group. Analyses were conducted on the combined sample. These analyses investigated the effects of age (adolescent vs emerging adult vs adult) on task performance (i.e., RT and accuracy).

**Table S10**

*Summary of H3 Analyses*

|  | **Reaction Time** | | | | **Accuracy** | | | |
| --- | --- | --- | --- | --- | --- | --- | --- | --- |
| *Predictors* | *df* | *F* | *p* | *R^2^m/R^2^c* | *df* | *F* | *p* | *R^2^m/R^2^c* |
| ***A:*** *Effects of Age on H1a* | | | | | | | | |
|  |  |  |  | 0.06/0.80 |  |  |  | 0.04/0.53 |
| Condition | 724.00 | 2.72 | .100 |  | **725.00** | **10.47** | **.001** |  |
| Age | **487.53** | **8.06** | **<.001** |  | **712.91** | **5.38** | **.005** |  |
| Condition x Age | 724.18 | 1.41 | .245 |  | 725.00 | 1.13 | .323 |  |
| ***B:*** *Effects of Age on H1b* | | | | | | | | |
|  |  |  |  | 0.06/0.80 |  |  |  | 0.05/0.54 |
| Condition | **721.00** | **5.30** | **.005** |  | **722.00** | **7.68** | **<.001** |  |
| Age | **484.73** | **8.09** | **<.001** |  | **702.94** | **5.43** | **.005** |  |
| Condition x Age | 721.09 | 1.16 | .328 |  | 722.00 | 0.63 | .639 |  |
| ***C:*** *Effects of Age on H1c (Social vs Non-Social Condition)* | | | | | | | | |
|  |  |  |  | 0.06/0.73 |  |  |  | 0.03/0.48 |
| Condition | 1808.58 | 0.03 | .857 |  | **1811.00** | **7.33** | **.007** |  |
| Valence | 1808.44 | 0.53 | .467 |  | 1811.00 | 3.12 | .078 |  |
| Age | 610.33 | 7.80 | **<.001** |  | **1044.40** | **7.70** | **<.001** |  |
| Condition x Valence | 1808.29 | 2.74 | .098 |  | 1811.00 | 0.02 | .892 |  |
| Condition x Age | 1808.41 | 1.48 | .227 |  | 1811.00 | 0.81 | .444 |  |
| Valence x Age | 1808.18 | 0.15 | .857 |  | 1811.00 | 1.87 | .154 |  |
| Condition x Valence x Age | 1808.12 | 0.13 | .875 |  | 1811.00 | 0.12 | .887 |  |
| ***D:*** *Effects of Age on H1c (Social-Self vs Social-Other vs Non-Social Condition)* | | | | | | | | |
|  |  |  |  | 0.07/0.75 |  |  |  | 0.04/0.49 |
| Condition | 1802.27 | 0.19 | .827 |  | **1805.00** | **5.05** | **.007** |  |
| Valence | 1802.41 | 0.57 | .452 |  | 1805.00 | 3.18 | .075 |  |
| Age | **593.68** | **7.92** | **<.001** |  | **1030.40** | **7.77** | **<.001** |  |
| Condition x Valence | **1802.14** | **9.96** | **<.001** |  | 1805.00 | 0.06 | .943 |  |
| Condition x Age | 1802.19 | 1.01 | .399 |  | 1805.00 | 0.49 | .740 |  |
| Valence x Age | 1802.17 | 0.16 | .849 |  | 1805.00 | 1.91 | .149 |  |
| Condition x Valence x Age | 1802.06 | 0.13 | .973 |  | 1805.00 | 0.43 | .785 |  |
| ***E:*** *Effects of Age on H2a* | | | | | | | | |
|  |  |  |  | 0.06/0.80 |  |  |  | 0.04/0.53 |
| Condition | 721.00 | 0.01 | .920 |  | 722.00 | 0.00 | .990 |  |
| Social sensitivity | 482.64 | 0.03 | .872 |  | 706.55 | 3.30 | .070 |  |
| Age | 483.74 | 1.23 | .293 |  | 706.55 | 1.02 | .361 |  |
| Condition x Social sensitivity | 721.00 | 0.59 | .444 |  | 722.00 | 1.71 | .191 |  |
| Condition x Age | 721.24 | 0.83 | .438 |  | 722.00 | 0.75 | .471 |  |
| Social sensitivity x Age | 484.77 | 0.19 | .830 |  | 706.55 | 2.95 | .053 |  |
| Condition x Social sensitivity x Age | 721.46 | 0.26 | .775 |  | 722.00 | 1.86 | .156 |  |
| ***F:*** *Effects of Age on H2b* | | | | | | | | |
|  |  |  |  | 0.07/0.80 |  |  |  | 0.05/0.55 |
| Condition | 715.00 | 0.40 | .671 |  | 716.00 | 0.01 | .994 |  |
| Social sensitivity | 480.34 | 0.03 | .872 |  | 697.33 | 3.33 | .068 |  |
| Age | 481.42 | 1.24 | .292 |  | 697.33 | 1.03 | .358 |  |
| Condition x Social sensitivity | 715.00 | 0.31 | .733 |  | 716.00 | 1.15 | .317 |  |
| Condition x Age | 715.12 | 0.52 | .719 |  | 716.00 | 0.48 | .749 |  |
| Social sensitivity x Age | 482.44 | 0.19 | .830 |  | 697.33 | 2.98 | .052 |  |
| Condition x Social sensitivity x Age | 715.22 | 0.16 | .960 |  | 716.00 | 1.09 | .363 |  |
| ***G:*** *Effects of Age on H2c (Social vs Non-Social Condition)* | | | | | | | | |
|  |  |  |  | 0.06/0.73 |  |  |  | 0.04/0.49 |
| Condition | 1800.35 | 0.83 | .361 |  | 1802.00 | 0.10 | .752 |  |
| Valence | 1800.02 | 0.11 | .741 |  | **1802.00** | **12.18** | **<.001** |  |
| Social sensitivity | 610.34 | 0.03 | .873 |  | 1028.50 | 0.01 | .939 |  |
| Age | 608.51 | 1.22 | .296 |  | 1028.50 | 1.37 | .255 |  |
| Condition x Valence | 1799.68 | 1.14 | .285 |  | 1802.00 | 0.21 | .644 |  |
| Condition x Social sensitivity | 1799.90 | 1.12 | .289 |  | 1802.00 | 0.56 | .454 |  |
| Valence x Social sensitivity | 1799.6 | 0.42 | .517 |  | **1802.00** | **9.30** | **.002** |  |
| Condition x Age | 1799.80 | 1.29 | .277 |  | 1802.00 | 0.05 | .947 |  |
| Valence x Age | 1799.44 | 0.05 | .948 |  | **1802.00** | **6.34** | **.002** |  |
| Social sensitivity x Age | 607.80 | 0.15 | .857 |  | 1028.50 | 0.47 | .623 |  |
| Condition x Valence x Social sensitivity | 1799.46 | 0.24 | .622 |  | 1802.00 | 0.31 | .581 |  |
| Condition x Valence x Age | 1799.30 | 0.41 | .661 |  | 1802.00 | 0.60 | .548 |  |
| Condition x Social sensitivity x Age | 1799.80 | 0.55 | .574 |  | 1802.00 | 0.32 | .729 |  |
| Valence x Social sensitivity x Age | 1799.29 | 0.03 | .969 |  | **1802.00** | **5.02** | **.007** |  |
| Condition x Valence x Social sensitivity x Age | 1799.19 | 0.32 | .728 |  | 1802.00 | 0.78 | .457 |  |
| ***H:*** *Effects of Age on H2c (Social-Self vs Social-Other vs Non-Social Condition)* | | | | | | | | |
|  |  |  |  | 0.08/0.75 |  |  |  | 0.05/0.50 |
| Condition | 1787.63 | 0.58 | .561 |  | 1790.00 | 0.15 | .863 |  |
| Valence | 1787.95 | 0.12 | .734 |  | **1790.00** | **12.40** | **<.001** |  |
| Social sensitivity | 593.91 | 0.03 | .872 |  | 1015.70 | 0.01 | .939 |  |
| Age | 592.20 | 1.24 | .291 |  | 1015.70 | 1.38 | .253 |  |
| Condition x Valence | 1787.32 | 0.68 | .509 |  | 1790.00 | 0.40 | .674 |  |
| Condition x Social sensitivity | 1787.42 | 0.91 | .402 |  | 1790.00 | 0.92 | .401 |  |
| Valence x Social sensitivity | 1787.64 | 0.45 | .504 |  | **1790.00** | **9.46** | **.002** |  |
| Condition x Age | 1787.38 | 1.04 | .387 |  | 1790.00 | 0.18 | .948 |  |
| Valence x Age | 1787.41 | 0.06 | .945 |  | **1790.00** | **6.51** | **.002** |  |
| Social sensitivity x Age | 591.54 | 0.16 | .855 |  | 1015.70 | 0.48 | .621 |  |
| Condition x Valence x Social sensitivity | 1787.21 | 0.92 | .401 |  | 1790.00 | 0.39 | .676 |  |
| Condition x Valence x Age | 1787.14 | 0.64 | .636 |  | 1790.00 | 0.34 | .851 |  |
| Condition x Social sensitivity x Age | 1787.38 | 0.50 | .738 |  | 1790.00 | 0.40 | .810 |  |
| Valence x Social sensitivity x Age | 1787.27 | 0.03 | .967 |  | **1790.00** | **5.11** | **.006** |  |
| Condition x Valence x Social sensitivity x Age | 1787.09 | 0.64 | .637 |  | 1790.00 | 0.51 | .731 |  |

*Note*. Bolded text indicates statistically significant effects. Analyses were conducted on the combined sample. H3 investigated whether the effects of condition (H1a: social vs non-social; H1b: social-self vs social-other vs non-social), valence (H1c), and social sensitivity (H2) on task performance (i.e., RT and accuracy) would differ as a function of age (adolescent vs emerging adult vs adult).

**Table S11**

*Summary of H4 Analyses*

|  | **Reaction Time** | | | | **Accuracy** | | | |
| --- | --- | --- | --- | --- | --- | --- | --- | --- |
| *Predictors* | *df* | *F* | *p* | *R^2^m/R^2^c* | *df* | *F* | *p* | *R^2^m/R^2^c* |
| ***A:*** *Effects of Personal Network Size* | | | | | | | | |
|  |  |  |  | 0.02/0.80 |  |  |  | 0.02/0.60 |
| Condition | **242.00** | **7.68** | **.006** |  | 242.00 | 0.72 | .395 |  |
| Size | 159.38 | 0.83 | .363 |  | 210.87 | 0.73 | .394 |  |
| Condition x Size | 242.00 | 0.37 | .545 |  | 242.00 | 3.06 | .081 |  |
| ***B:*** *Effects of Personal Network Quality* | | | | | | | | |
|  |  |  |  | 0.01/0.80 |  |  |  | 0.02/0.60 |
| Condition | **242.00** | **5.03** | **.026** |  | **242.00** | **5.93** | **.016** |  |
| Quality | 159.23 | 0.54 | .465 |  | 213.40 | 1.40 | .238 |  |
| Condition x Quality | 242.00 | 0.33 | .568 |  | 242.00 | 0.00 | .989 |  |
| ***C:*** *Effects of Personal Network Satisfaction* | | | | | | | | |
|  |  |  |  | 0.01/0.80 |  |  |  | 0.04/0.59 |
| Condition | **244.00** | **4.94** | **.027** |  | 244.00 | 0.33 | .568 |  |
| Satisfaction | 159.63 | 0.16 | .692 |  | **218.03** | **4.60** | **.033** |  |
| Condition x Satisfaction | 244.00 | 1.40 | .237 |  | 244.00 | 0.23 | .635 |  |
| ***D:*** *Effects of Cool Ratio* | | | | | | | | |
|  |  |  |  | 0.03/0.80 |  |  |  | 0.07/0.61 |
| Condition | **230.00** | **10.06** | **.002** |  | **230.00** | **6.19** | **.014** |  |
| Cool Ratio | 152.00 | 2.53 | .113 |  | **204.64** | **14.14** | **<.001** |  |
| Condition x Cool Ratio | 230.00 | 0.04 | .836 |  | **230.00** | **4.61** | **.033** |  |

*Note*. Bolded text indicates statistically significant effects. Size = sum of total number of in person and online friends; Quality = sum of total number of friends participants could ask a favour of and would trust to keep a secret; Satisfaction = sum of reported happiness with how often participants spend time with friends online and in person; Cool ratio = ratio of the number of people participants follow on social media compared to the number of people that follow participants on social media. Analyses were conducted on the adolescent sample. H4 investigated whether the effects of condition (social vs non-social) on task performance (i.e., RT and accuracy) would be associated with improved social functioning (i.e., increased network size, quality, satisfaction, and cool ratio).

**Figure S3**

*Network Task Performance Depending on Cool Ratio and Condition*

*
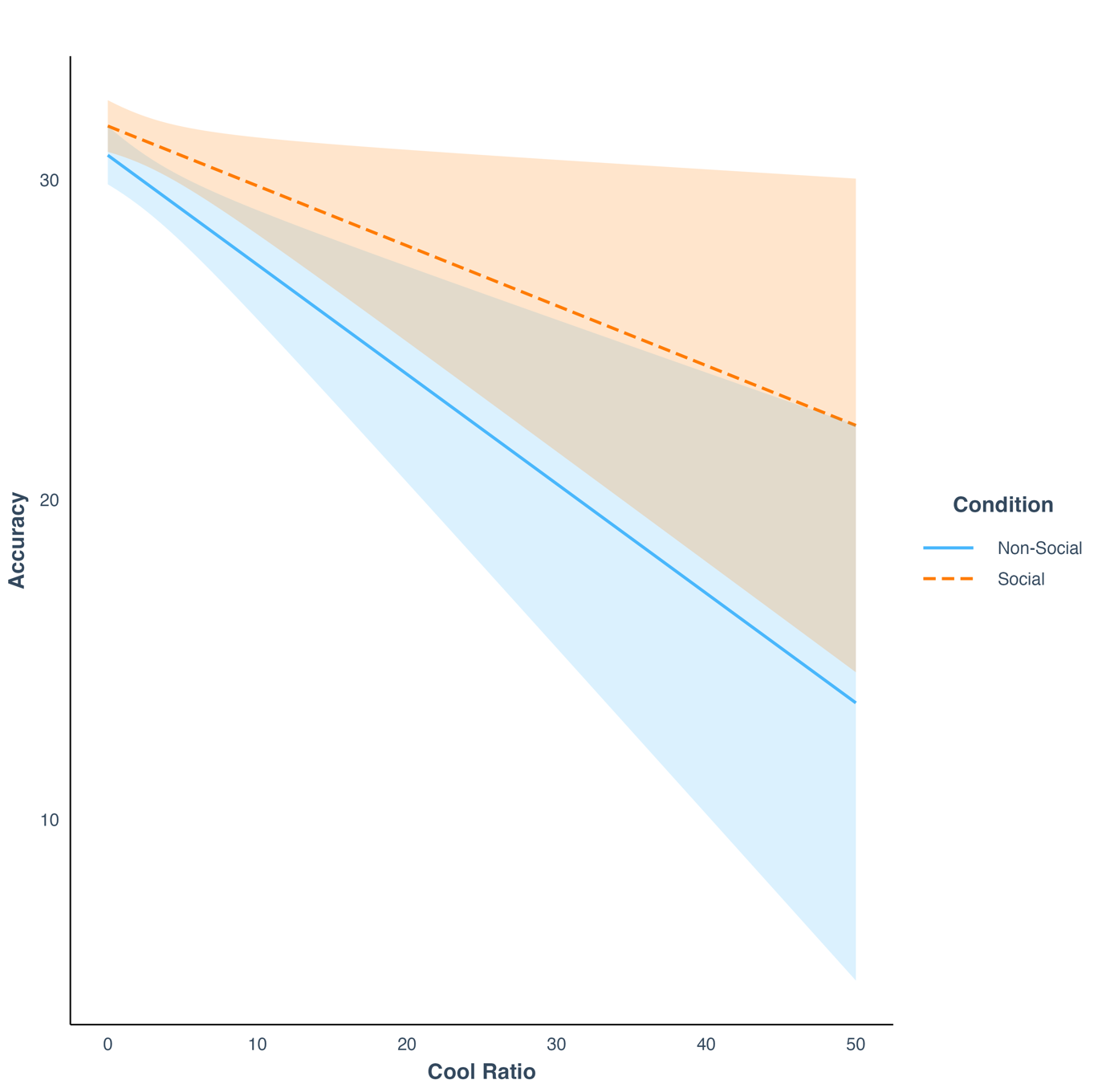
*

*Note*. Interaction effects between cool ratio (ratio of the number of people participants follow on social media compared to the number of people that follow participants on social media) and condition (non-social vs social) on participant accuracy in the network task. Accuracy is operationalised as number of total correct trials out of 36.

**Table S12**

*Summary of Exploratory Cognitive Load Analyses.*

|  | **Reaction Time** | | | | **Accuracy** | | | |
| --- | --- | --- | --- | --- | --- | --- | --- | --- |
| *Predictors* | *df* | *F* | *p* | *R^2^m/R^2^c* | *df* | *F* | *p* | *R^2^m/R^2^c* |
|  |  |  |  | 0.12/0.70 |  |  |  | 0.05/0.51 |
| Load | **614.00** | **306.73** | **<.001** |  | **614.00** | **77.21** | **<.001** |  |

*Note*. Bolded text indicates statistically significant effects. Load indicates cognitive load in the network task trials (high vs low). Analyses were conducted on the adolescent sample. These analyses investigated the effect of cognitive load (high vs low) on task performance (i.e., RT and accuracy).

**Figure S4**

*Network Task Performance Across Low and High Cognitive Load*

*
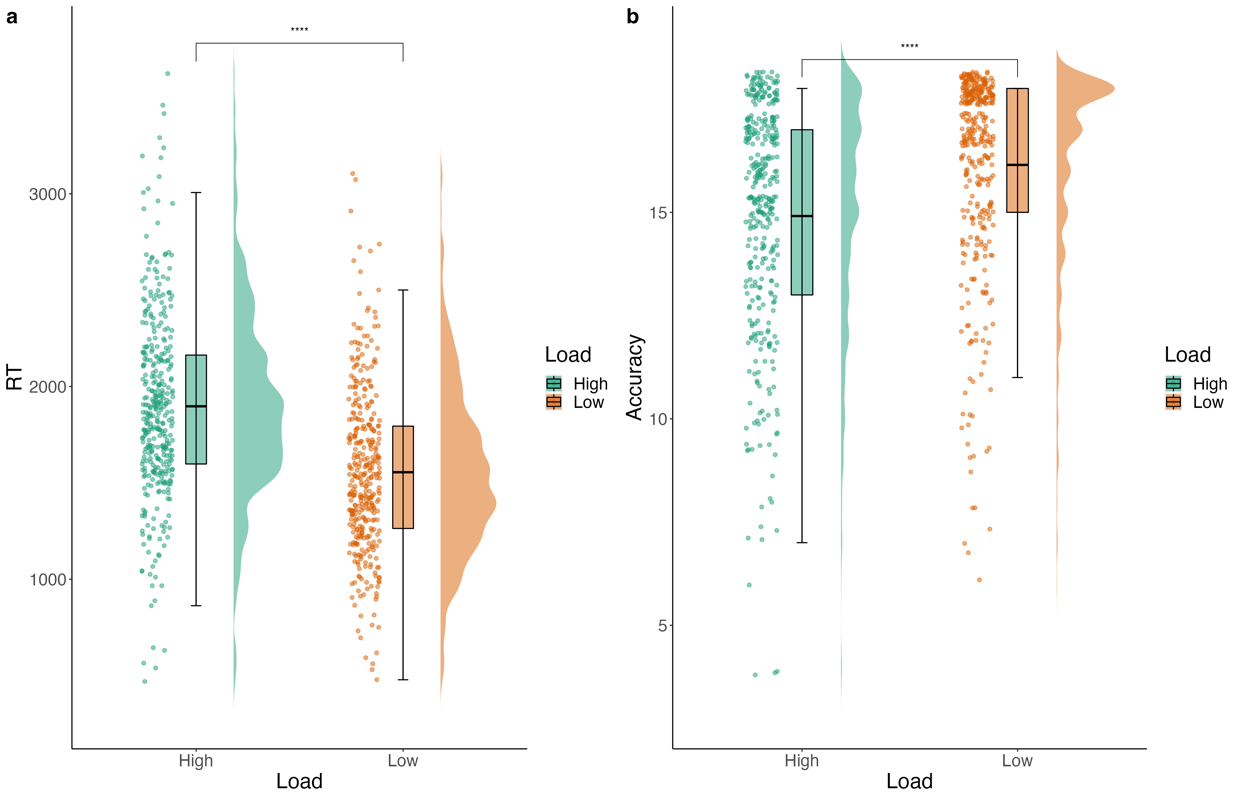
*

*Note.* **a.** Participant reaction time (RT) collapsed across all network task conditions (social-self, social-other, non-social) under different loads (high = 4 people/cities; low = 3 people/cities). Reaction time is operationalised as average reaction time on correct trials of the network task. **b.** Participant accuracy collapsed across all network task conditions (social-self, social-other, non-social) under different loads (high = 4 people/cities; low = 3 people/cities). Accuracy is operationalised as number of total correct trials out of 18 (36 trials divided into high vs low load trials).

**Table S13**

*Summary of Exploratory Cognitive Load and Condition Analyses (H1a and H1b)*

|  | **Reaction Time** | | | | **Accuracy** | | | |
| --- | --- | --- | --- | --- | --- | --- | --- | --- |
| *Predictors* | *df* | *F* | *p* | *R^2^m/R^2^c* | *df* | *F* | *p* | *R^2^m/R^2^c* |
| ***A:*** *Effects of Load on H1a* | | | | | | | | |
|  |  |  |  | 0.13/0.71 |  |  |  | 0.07/0.53 |
| Condition | **612.00** | **20.05** | **<.001** |  | **612.00** | **24.34** | **<.001** |  |
| Load | **612.00** | **153.04** | **<.001** |  | **612.00** | **59.26** | **<.001** |  |
| Condition x Load | **612.00** | **6.65** | **.010** |  | **612.00** | **9.62** | **.002** |  |
| ***B:*** *Effects of Load on H1b* | | | | | | | | |
|  |  |  |  | 0.16/0.74 |  |  |  | 0.08/0.55 |
| Condition | **610.00** | **16.72** | **<.001** |  | **610.00** | **13.10** | **<.001** |  |
| Load | **610.00** | **171.24** | **<.001** |  | **610.00** | **61.48** | **<.001** |  |
| Condition x Load | **610.00** | **35.67** | **<.001** |  | **610.00** | **13.61** | **<.001** |  |
|  | **Reaction Time** | | | | **Accuracy** | | | |
| *Predictors* | *β* | *95% CI* | *t* | *p* | *β* | *95% CI* | *t* | *p* |
| ***C:*** *Contrast Models for Effect of Load on H1b* | | | | | | | | |
| Non (High vs Low) | **0.85** | **0.67 - 1.03** | **13.09** | **<.001** | **−0.68** | **−0.92 - −0.44** | **-7.84** | **<.001** |
| Other (High vs Low) | **0.27** | **0.09 - 0.45** | **4.09** | **<.001** | −0.09 | −0.33 - 0.15 | -1.04 | .960 |
| Self (High vs Low) | **1.00** | **0.82 - 1.18** | **15.40** | **<.001** | **−0.60** | **−0.83 - −0.36** | **-6.91** | **<.001** |

*Note*. Bolded text indicates statistically significant effects. Non indicates non-social condition; Other indicates social-other condition; Self indicates social-self condition. Analyses were conducted on the adolescent sample. These analyses investigated whether the effect of condition (H1a: social vs non-social; H1b: social-self vs social-other vs non-social) on task performance (i.e., RT and accuracy) would differ as a function of cognitive load (high vs low).

**Figure S5**

*Network Task Performance Depending on Load and Condition*

**
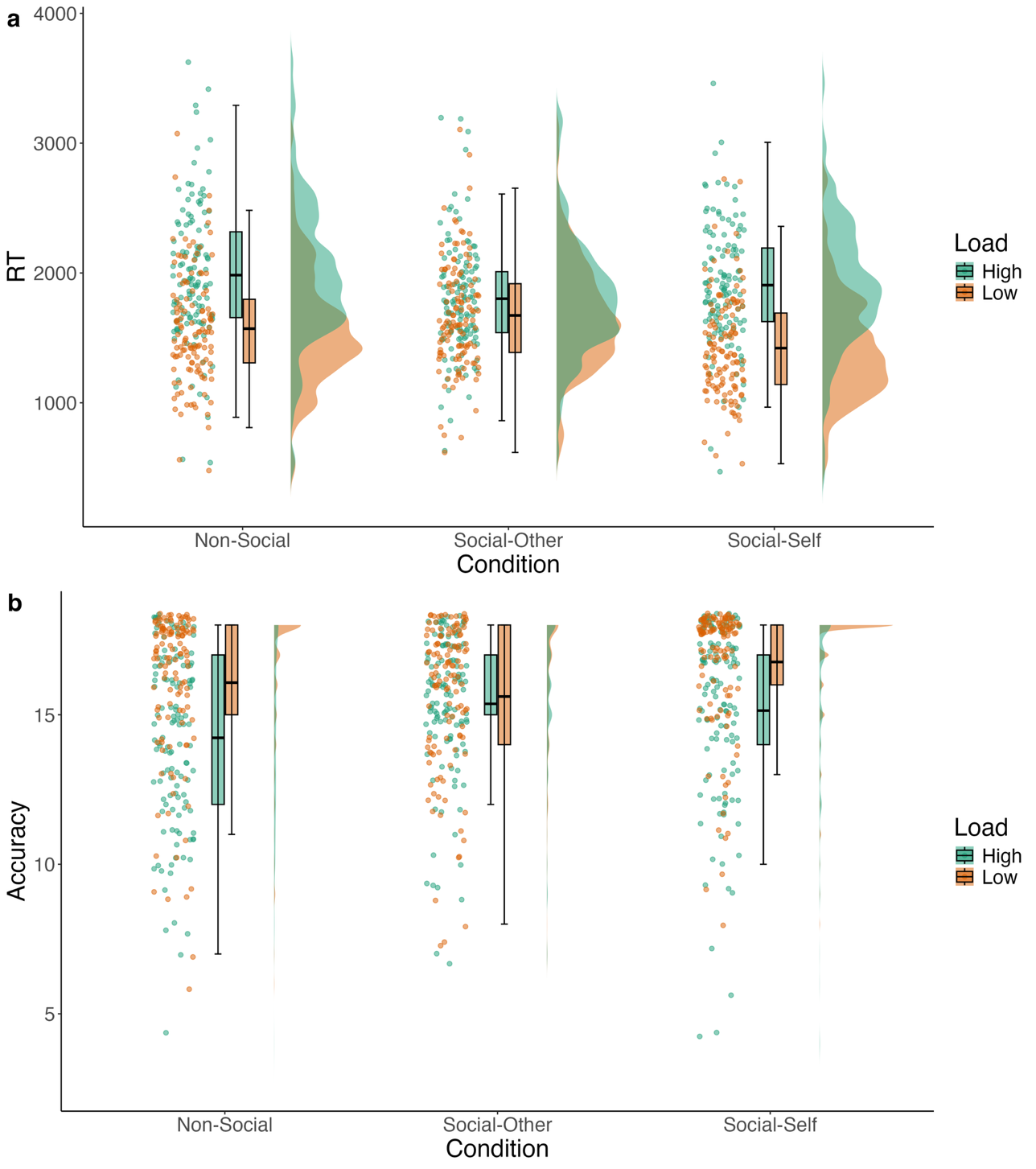
**

*Note*. **a.** Interaction effects between load (high vs low) and condition (non-social vs social-other vs social-self) on participant reaction time (RT). RT is operationalised as average reaction time on correct trials of the network task. **b.** Interaction effects between load (high vs low) and condition (non-social vs social-other vs social-self) on participant accuracy. Accuracy is operationalised as number of total correct trials out of 18 (36 trials divided into high vs low load trials).

**Table S14**

*Summary of Exploratory Preliminary Age (Continuous) Analyses*

|  | **Reaction Time** | | | | **Accuracy** | | | |
| --- | --- | --- | --- | --- | --- | --- | --- | --- |
| *Predictors* | *df* | *F* | *p* | *R^2^m/R^2^c* | *df* | *F* | *p* | *R^2^m/R^2^c* |
|  |  |  |  | 0.05/0.78 |  |  |  | 0.00/0.50 |
| Age | **361.96** | **23.09** | **<.001** |  | 362.00 | 0.36 | .548 |  |

*Note*. Bolded text indicates statistically significant effects. Analyses were conducted on the combined sample. These analyses investigated the effects of age (continuous) on task performance (i.e., RT and accuracy).

**Figure S6**

*Network Task Performance Depending on Age (Continuous)*

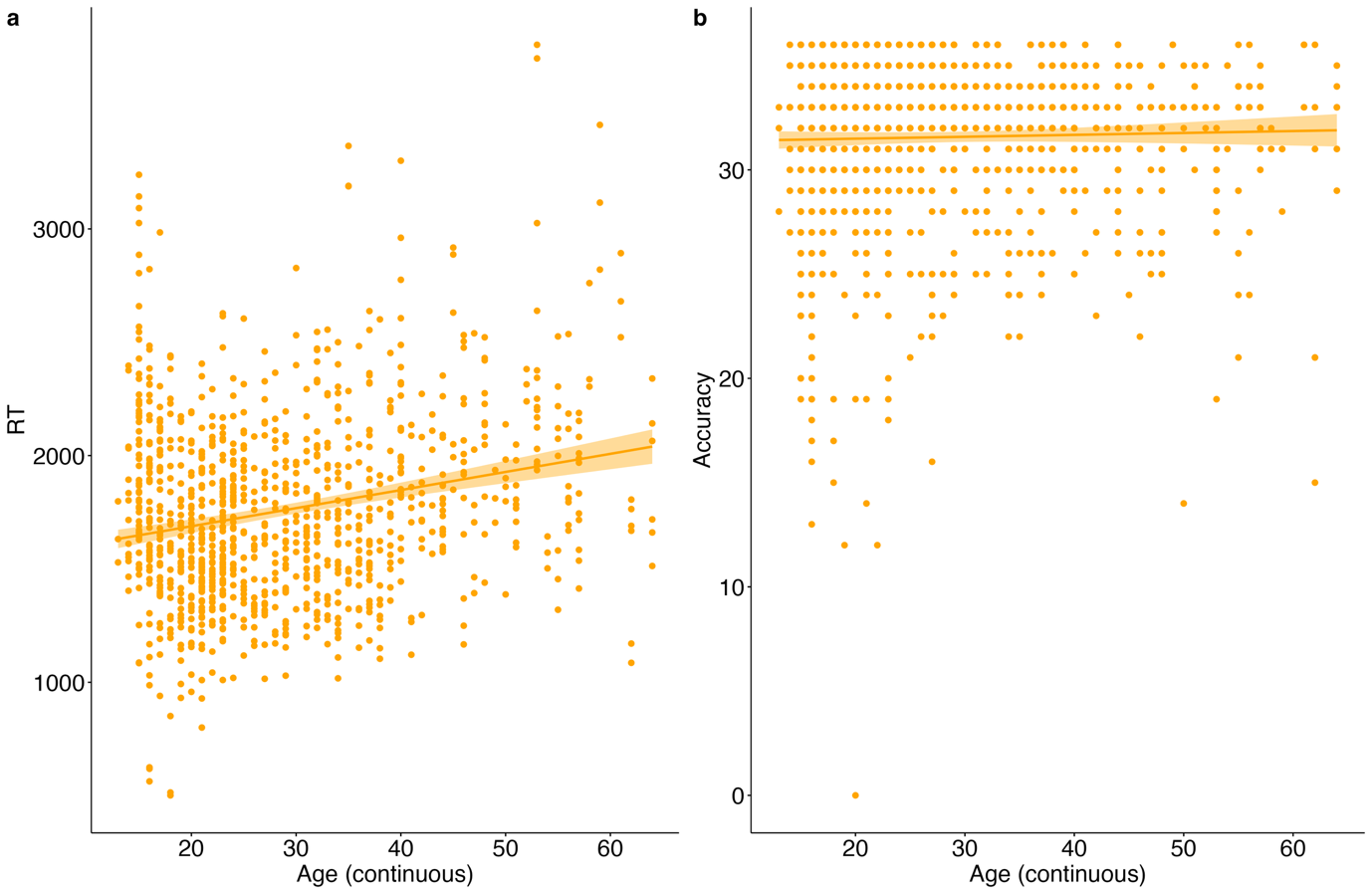


*Note*. **a,** Participant reaction time (RT) across age (continuous). Reaction time is operationalised as average reaction time on correct trials of the network task. **b**, Participant accuracy across age (continuous). Accuracy is operationalised as number of total correct trials out of 36 trials total.

**Table S15**

*Summary of Exploratory H3 Analyses (Age Continuous)*

|  | **Reaction Time** | | | | **Accuracy** | | | |
| --- | --- | --- | --- | --- | --- | --- | --- | --- |
| *Predictors* | *df* | *F* | *p* | *R^2^m/R^2^c* | *df* | *F* | *p* | *R^2^m/R^2^c* |
| ***A:*** *Effects of Age on H1a* | | | | | | | | |
|  |  |  |  | 0.06/0.79 |  |  |  | 0.02/0.53 |
| Condition | **725.19** | **7.45** | **.006** |  | **726.00** | **8.31** | **.004** |  |
| Age | **489.44** | **20.30** | **<.001** |  | 706.47 | 0.77 | .379 |  |
| Condition x Age | 725.09 | 0.03 | .857 |  | 726.00 | 0.46 | .498 |  |
| ***B:*** *Effects of Age on H1b* | | | | | | | | |
|  |  |  |  | 0.07/0.80 |  |  |  | 0.03/0.54 |
| Condition | **723.09** | **7.09** | **.001** |  | **724.00** | **5.90** | **.003** |  |
| Age | **486.57** | **20.37** | **<.001** |  | 696.42 | 0.78 | .377 |  |
| Condition x Age | 723.05 | 0.60 | .548 |  | 724.00 | 0.24 | .784 |  |
| ***C:*** *Effects of Age on H1c (Social vs Non-Social Condition)* | | | | | | | | |
|  |  |  |  | 0.06/0.73 |  |  |  | 0.01/0.48 |
| Condition | 1811.36 | 3.55 | .060 |  | **1814.00** | **4.45** | **.035** |  |
| Valence | 1811.14 | 0.09 | .809 |  | 1814.00 | 0.35 | .555 |  |
| Age | **611.44** | **14.83** | **<.001** |  | 1030.60 | 1.21 | .272 |  |
| Condition x Valence | 1811.10 | 0.54 | .463 |  | 1814.00 | 0.30 | .585 |  |
| Condition x Age | 1811.21 | 0.09 | .760 |  | 1814.00 | 0.23 | .634 |  |
| Valence x Age | 1811.09 | 0.72 | .397 |  | 1814.00 | 0.34 | .560 |  |
| Condition x Valence x Age | 1811.06 | 0.17 | .683 |  | 1814.00 | 0.02 | .876 |  |
| ***D:*** *Effects of Age on H1c (Social-Self vs Social-Other vs Non-Social Condition)* | | | | | | | | |
|  |  |  |  | 0.07/0.75 |  |  |  | 0.02/0.49 |
| Condition | 1807.20 | 2.32 | .099 |  | **1810.00** | **4.35** | **.013** |  |
| Valence | 1807.10 | 0.06 | .803 |  | 1810.00 | 0.36 | .551 |  |
| Age | **594.70** | **15.06** | **<.001** |  | 1016.20 | 1.22 | .270 |  |
| Condition x Valence | **1807.00** | **9.28** | **<.001** |  | 1810.00 | 0.40 | .673 |  |
| Condition x Age | 1807.10 | 0.15 | .863 |  | 1810.00 | 0.52 | .593 |  |
| Valence x Age | 1807.10 | 0.77 | .381 |  | 1810.00 | 0.35 | .556 |  |
| Condition x Valence x Age | 1807.00 | 0.24 | .789 |  | 1810.00 | 1.00 | .366 |  |
| ***E:*** *Effects of Age on H2a* | | | | | | | | |
|  |  |  |  | 0.06/0.80 |  |  |  | 0.03/0.53 |
| Condition | **723.19** | **5.01** | **.026** |  | 724.00 | 0.06 | .803 |  |
| Social sensitivity | 487.54 | 0.32 | .573 |  | **707.29** | **6.56** | **.011** |  |
| Age | 486.11 | 2.74 | .099 |  | 707.29 | 3.57 | .059 |  |
| Condition x Social sensitivity | 723.41 | 1.76 | .185 |  | 724.00 | 0.97 | .325 |  |
| Condition x Age | 723.11 | 1.66 | .198 |  | 724.00 | 0.14 | .707 |  |
| Social sensitivity x Age | 486.63 | 0.20 | .655 |  | **707.29** | **6.50** | **.011** |  |
| Condition x Social sensitivity x Age | 723.22 | 2.58 | .109 |  | 724.00 | 0.46 | .499 |  |
| ***F:*** *Effects of Age on H2b* | | | | | | | | |
|  |  |  |  | 0.07/0.80 |  |  |  | 0.04/0.54 |
| Condition | 719.09 | 2.57 | .077 |  | 720.00 | 0.26 | .771 |  |
| Social sensitivity | 484.79 | 0.32 | .572 |  | **696.66** | **6.63** | **.010** |  |
| Age | 483.39 | 2.75 | .098 |  | 696.66 | 3.61 | .058 |  |
| Condition x Social sensitivity | 719.20 | 1.45 | .236 |  | 720.00 | 1.80 | .166 |  |
| Condition x Age | 719.05 | 0.95 | .388 |  | 720.00 | 1.22 | .295 |  |
| Social sensitivity x Age | 483.90 | 0.20 | .655 |  | **696.66** | **6.57** | **.011** |  |
| Condition x Social sensitivity x Age | 719.11 | 1.88 | .154 |  | 720.00 | 1.57 | .208 |  |
| ***G:*** *Effects of Age on H2c (Social vs Non-Social Condition)* | | | | | | | | |
|  |  |  |  | 0.06/0.73 |  |  |  | 0.03/0.48 |
| Condition | 1805.58 | 2.42 | .120 |  | 1808.00 | 0.22 | .641 |  |
| Valence | 1805.31 | 0.48 | .488 |  | **1808.00** | **5.52** | **.019** |  |
| Social sensitivity | 609.44 | 0.68 | .411 |  | 1032.00 | 1.24 | .265 |  |
| Age | 607.69 | 1.71 | .192 |  | 1032.00 | 0.28 | .597 |  |
| Condition x Valence | 1805.21 | 0.22 | .636 |  | 1808.00 | 0.13 | .724 |  |
| Condition x Social sensitivity | 1805.61 | 0.82 | .367 |  | 1808.00 | 0.13 | .716 |  |
| Valence x Social sensitivity | 1805.18 | 0.46 | .498 |  | **1808.00** | **5.48** | **.019** |  |
| Condition x Age | 1805.36 | 1.05 | .306 |  | 1808.00 | 0.02 | .902 |  |
| Valence x Age | 1805.20 | 0.25 | .614 |  | **1808.00** | **5.28** | **.022** |  |
| Social sensitivity x Age | 606.97 | 0.17 | .684 |  | 1032.00 | 1.38 | .241 |  |
| Condition x Valence x Social sensitivity | 1805.12 | 0.07 | .798 |  | 1808.00 | 0.48 | .488 |  |
| Condition x Valence x Age | 1805.13 | 0.04 | .849 |  | 1808.00 | 0.41 | .525 |  |
| Condition x Social sensitivity x Age | 1805.33 | 1.02 | .312 |  | 1808.00 | 0.00 | .998 |  |
| Valence x Social sensitivity x Age | 1805.10 | 0.01 | .911 |  | **1808.00** | **4.90** | **.027** |  |
| Condition x Valence x Social sensitivity x Age | 1805.06 | 0.24 | .623 |  | 1808.00 | 0.68 | .408 |  |
| ***H:*** *Effects of Age on H2c (Social-Self vs Social-Other vs Non-Social Condition)* | | | | | | | | |
|  |  |  |  | 0.07/0.75 |  |  |  | 0.04/0.49 |
| Condition | 1797.27 | 1.87 | .154 |  | 1800.0 | 0.20 | .820 |  |
| Valence | 1797.29 | 0.51 | .474 |  | **1800.0** | **5.64** | **.018** |  |
| Social sensitivity | 592.85 | 0.69 | .408 |  | 1017.1 | 1.26 | .263 |  |
| Age | 591.21 | 1.73 | .189 |  | 1017.1 | 0.28 | .595 |  |
| Condition x Valence | 1797.10 | 1.40 | .248 |  | 1800.0 | 0.09 | .913 |  |
| Condition x Social sensitivity | 1797.29 | 0.75 | .472 |  | 1800.0 | 1.06 | .346 |  |
| Valence x Social sensitivity | 1797.16 | 0.49 | .485 |  | **1800.0** | **5.60** | **.018** |  |
| Condition x Age | 1797.17 | 0.70 | .495 |  | 1800.0 | 0.33 | .719 |  |
| Valence x Age | 1797.19 | 0.27 | .603 |  | **1800.0** | **5.40** | **.020** |  |
| Social sensitivity x Age | 590.54 | 0.17 | .681 |  | 1017.1 | 1.39 | .239 |  |
| Condition x Valence x Social sensitivity | 1797.06 | 0.05 | .950 |  | 1800.0 | 0.25 | .782 |  |
| Condition x Valence x Age | 1797.06 | 0.02 | .976 |  | 1800.0 | 0.46 | .630 |  |
| Condition x Social sensitivity x Age | 1797.15 | 0.89 | .410 |  | 1800.0 | 0.89 | .410 |  |
| Valence x Social sensitivity x Age | 1797.09 | 0.01 | .908 |  | **1800.0** | **5.00** | **.025** |  |
| Condition x Valence x Social sensitivity x Age | 1797.03 | 0.14 | .867 |  | 1800.0 | 0.35 | .701 |  |

*Note*. Bolded text indicates statistically significant effects. Analyses were conducted on the combined sample. These analyses investigated whether the effects of condition (H1a: social vs non-social; H1b: social-self vs social-other vs non-social), valence (H1c), and social sensitivity (H2) on task performance (i.e., RT and accuracy) would differ as a function of age (continuous).

**References**

Andrews, J. L., Grunewald, K., & Schweizer, S. (2024). A human working memory advantage for social network information. *Proceedings of the Royal Society B*, *291*, 20241930. <https://doi.org/10.1098/rspb.2024.1930>

Andrews, J. L., Khin, A. C., Crayn, T., Humphreys, K., & Schweizer, S. (2022). Measuring online and offline social rejection sensitivity in the digital age. *Psychological Assessment*, No Pagination Specified-No Pagination Specified. https://doi.org/10.1037/pas0001136

Bartoń, K. (2010). *MuMIn: Multi-Model Inference* (p. 1.47.5) [Dataset]. https://doi.org/10.32614/CRAN.package.MuMIn

Bates, D., Mächler, M., Bolker, B., & Walker, S. (2015). Fitting linear mixed-effects models using lme4. *Journal of Statistical Software*, *67*, 1–48.

Lenth, R. V. (2017). Using lsmeans. *Journal of Statistical Software*, *69*, 1–33.

Lovibond, P. F., & Lovibond, S. H. (1995). The structure of negative emotional states: Comparison of the Depression Anxiety Stress Scales (DASS) with the Beck Depression and Anxiety Inventories. *Behaviour Research and Therapy*, *33*(3), 335–343. https://doi.org/10.1016/0005-7967(94)00075-U

Stanley, D., & Stanley, M. D. (2018). *Package ‘apaTables.’*
